# Costs incurred by people receiving tuberculosis treatment in low-income and middle-income countries: a meta-regression analysis

**DOI:** 10.1016/S2214-109X(23)00369-8

**Published:** 2023-09-19

**Authors:** Allison Portnoy, Takuya Yamanaka, Peter Nguhiu, Nobuyuki Nishikiori, Ineés Garcia Baena, Katherine Floyd, Nicolas A Menzies

**Affiliations:** a Department of Global Health, Boston University School of Public Health, Boston, MA, USA; b Center for Health Decision Science, Harvard TH Chan School of Public Health, Boston, MA, USA; c Department of Global Health and Population, Harvard TH Chan School of Public Health, Boston, MA, USA; d Global TB Programme, World Health Organization, Geneva, Switzerland

## Abstract

**Background:**

People accessing and completing treatment for tuberculosis can face large economic costs, even when treatment is provided free of charge. The WHO End TB Strategy targets the elimination of catastrophic costs among tuberculosis-affected households. While low-income and middle-income countries (LMICs) represent 99% of global tuberculosis cases, only 29 of 135 LMICs had conducted national surveys of costs for patients with tuberculosis by December, 2022. We estimated costs for patients with tuberculosis in countries that have not conducted a national survey, to provide evidence on the economic burden of tuberculosis in these settings and inform estimates of global economic burden.

**Methods:**

We extracted data from 22 national surveys of costs faced by patients with tuberculosis that were completed across 2015–22 and met inclusion criteria. Using a Bayesian meta-regression approach, we used these data and covariate data for all 135 LMICs to estimate per-patient costs (2021 US$) by cost category (ie, direct medical, direct non-medical, and indirect), country, drug resistance, and household income quintile. We also estimated the proportion of households experiencing catastrophic total costs (defined as >20% of annual household income) as a result of tuberculosis disease.

**Findings:**

Across LMICs, mean direct medical costs incurred by patients with tuberculosis were estimated as US$211 (95% uncertainty interval 154–302), direct non-medical costs were $512 (428–620), and indirect costs were $530 (423–663) per episode of tuberculosis. Overall, per-patient costs were $1253 (1127–1417). Estimated proportions of tuberculosis-affected households experiencing catastrophic total costs ranged from 75·2% (70·3–80·0) in the poorest quintile to 42·5% (34·3–51·5) in the richest quintile, compared with 54·9% (47·0–63·2) overall.

**Interpretation:**

Tuberculosis diagnosis and treatment impose substantial costs on affected households. Eliminating these economic losses is crucial for removing barriers to accessing tuberculosis diagnosis and completing treatment among affected households and achieving the targets set in WHO's End TB Strategy.

**Funding:**

World Health Organization.

## Introduction

Tuberculosis causes substantial disability and mortality in many low-income and middle-income countries (LMICs), and for the majority of the past 20 years was the leading global cause of death from a single infectious agent.^1^ In 2021, an estimated 1·6 million people died from tuberculosis (including 0·2 million who were HIV positive).^1^ Many people who develop tuberculosis disease experience substantial economic costs during the disease episode.^2^, ^3^ These result from reduced productive activity during the period of illness and treatment, as well as additional out-of-pocket expenditures associated with tuberculosis care. Even though tuberculosis treatment is provided free of charge in most countries, many studies have documented challenges faced by individuals seeking care for their symptoms, with sometimes multiple missed diagnoses and incorrect treatments provided before an accurate diagnosis is made.^4^, ^5^, ^6^, ^7^, ^8^, ^9^ Moreover, effective tuberculosis treatment typically requires regimens of 6 months—and often longer for drug-resistant disease—such that the cumulative non-medical costs faced to access care (eg, travel costs to reach the treatment clinic) can be substantial. Some patients also report having to pay for aspects of their care, despite national policies for free publicly provided treatment. The combined effect of reduced ability to work and high treatment costs can have major implications for the households of patients, as the large majority of people with tuberculosis are working-age adults who predominantly come from poor households with limited resources to cope with interruptions in income and unanticipated health-care expenditures.^1^

Reducing costs faced by patients and their households is essential to ensure that everyone with tuberculosis can access care and complete treatment, and in turn to minimise the number of people dying unnecessarily from tuberculosis. The WHO End TB Strategy (2016–35) includes milestones and targets for three high-level indicators: tuberculosis incidence (new cases per 100 000 population per year), the absolute number of tuberculosis deaths per year, and the percentage of patients with tuberculosis and their households facing catastrophic total costs as a result of tuberculosis disease.^10^, ^11^ For the latter, the first milestone (for 2020) was that no tuberculosis-affected households face catastrophic total costs, to be sustained thereafter. The WHO Global Tuberculosis Programme has developed standard methods for measuring costs faced by patients and their households, based on national facility-based surveys.^12^ By the end of 2022, surveys had been completed in 29 countries.^1^, ^9^ These can be used to inform policy and planning to mitigate costs, including through general health financing policy (eg, national health insurance schemes and benefit packages) and social protection. However, countries with survey data represent a small subset of countries with a high burden of tuberculosis.

**Research in context**

**Evidence before this study**

National surveys of costs faced by patients with tuberculosis and their households that used methods recommended by WHO have been conducted in 29 low-income and middle-income countries (LMICs) with a high burden of tuberculosis as of December, 2022. Available results from these surveys showed that people with tuberculosis experience high out-of-pocket expenditures and indirect costs (eg, income losses), and that these costs represent a large share of household income, especially in poorer income quintiles. The percentage of patients with tuberculosis and their households facing catastrophic total costs ranged from 13% to 92%, far from the target of zero set in WHO's End TB Strategy.

We searched MEDLINE on July 19, 2023, using the terms (“tuberculosis” AND “cost” AND “patient”) for articles published with English abstracts and describing costs for patients with tuberculosis published as of July 11, 2023. Available studies reported analyses based on individual cost study samples or reported results for several surveys. No available studies combined data from multiple studies to make estimates for countries that have not yet been able to complete surveys. Estimates of patient-level tuberculosis costs are unavailable for as many as 108 of 135 LMICs.

**Added value of this study**

This study is the first to provide estimates of costs faced by patients with tuberculosis and their households for all LMICs. The analysis relied on Bayesian meta-regression methods to predict these costs, as well as the national proportion of households experiencing catastrophic total costs due to tuberculosis, to fill the knowledge gap resulting from a scarcity of national surveys conducted in all LMICs.

**Implications of all the available evidence**

Patients with tuberculosis and their households face substantial economic barriers to diagnosis and treatment in all LMICs, and the WHO End TB Strategy target that no tuberculosis-affected households face catastrophic total costs has probably not been achieved in any LMIC. Actions to mitigate and eliminate these cost barriers are required in all LMICs. Our modelled estimates could be useful to inform these actions in countries that have yet to conduct a national survey.

Using data from published national surveys, we estimated costs faced by tuberculosis-affected households in 135 LMICs, representing 98·8% of the global tuberculosis burden.^13^ We aim to report estimates of per-episode costs at the country level, both overall and stratified by cost category, income quintile, and by diagnosed tuberculosis drug resistance. We also aim to report estimates of the percentage of households in each country experiencing catastrophic total costs due to tuberculosis (defined as costs of >20% of household income or expenditure), both overall and by income quintile.

## Methods

We developed a statistical approach to predict the costs experienced by patients treated for tuberculosis in LMICs, as well as the national proportion of households experiencing catastrophic total costs due to tuberculosis. Estimates were calculated using data from nationally representative cost surveys conducted among patients with tuberculosis in a sample of countries.^9^ This study was exempt from institutional ethics approval because it analysed secondary data that are not classified as human participants research.

### Patient cost survey data

We considered countries classified as low-income or middle-income according to the World Bank country income classification in 2021.^14^ Between 2015 and 2022, 29 LMICs conducted national surveys of costs faced by patients with tuberculosis and their households,^9^ using standardised methods developed by the WHO Global TB Programme.^12^ These surveys collected representative data on direct medical costs, direct non-medical costs, and indirect costs incurred during the tuberculosis disease episode, as well as basic clinical and demographic information and household income (based on self-reporting of either annual household income [preferred] or expenditure).^12^ Direct medical costs included costs incurred during pre-diagnosis, during hospitalisation or a medical visit (bed day charges, consultation fees, radiography, medicines, laboratory tests, and other procedures), a directly observed treatment visit, or a visit to pick up tuberculosis drugs. Direct non-medical costs included the cost of transportation to and from a medical visit and the cost of food that the patient (and their accompanying household members) had to purchase while travelling to a health facility (including the cost of overnight accommodation if required). Indirect costs are measured through the income that patients reported losing during treatment, or through a measure of the opportunity cost for seeking or being in care that is a valuation of time lost for the patient and their household members throughout the tuberculosis episode. The surveys used a cross-sectional, facility-based design for all patients registered for tuberculosis treatment in facilities linked to the national tuberculosis programme who attended a sampled facility during the study period. Survey participants reported on tuberculosis care-related expenditures and time spent seeking and receiving care, including the period before tuberculosis diagnosis. Costs incurred after treatment completion were not included. An overview of the cross-sectional design and analytical approach for these surveys is provided in the appendix (p 2).

This study included data from 22 of the individual country-representative patient cost surveys conducted by country tuberculosis programmes, with the support of the WHO Global TB Programme (table 1). This represents all surveys conducted using WHO's standardised approach before Feb 1, 2023, excluding seven countries for which either data could not be shared for the analysis or data were not available with the level of disaggregation required for the analysis.

**Table 1:** Low-income and middle-income country list

	**Income level**	**Survey year**	**Income measure**	**Per-capita gross domestic product (2021)**
Brazil	UMIC	2020	Reported income	$7510
Burkina Faso	LIC	2020	Reported income	$893
Colombia	UMIC	2022	Reported income	$6100
Democratic Republic of the Congo	LIC	2019	Reported expenditure as proxy	$577
Fiji	UMIC	2017	Reported income	$4650
Ghana	LMIC	2016	Reported income	$2360
Indonesia	LMIC	2020	Reported income	$4330
Kenya	LMIC	2017	Reported expenditure as proxy	$2080
Laos	LMIC	2018	Reported income	$2540
Mali	LIC	2021	Reported income	$874
Mongolia	LMIC	2018	Reported income	$4570
Myanmar	LMIC	2016	Reported income	$1210
Niger	LIC	2021	Reported income	$591
Nigeria	LMIC	2017	Reported income	$2070
Papua New Guinea	LMIC	2018	Reported income	$2670
Philippines	LMIC	2017	Reported income	$3460
Solomon Islands	LMIC	2019	Reported income	$2300
Tanzania	LMIC	2019	Reported expenditure as proxy	$1100
Thailand	UMIC	2021	Reported expenditure as proxy	$7070
Uganda	LIC	2017	Reported income	$884
Viet Nam	LMIC	2016	Reported income	$3760
Zimbabwe	LMIC	2018	Reported income	$1770

LIC defined as a gross national income per capita of US$1085 or less, LMIC defined as a gross national income per capita of $1086 to $4225, and UMIC defined as a gross national income per capita of $4226 to $13 205.^14^ UMIC=upper-middle-income country. LIC=low-income country. LMIC=lower-middle-income country.

We obtained summary statistics (ie, arithmetic mean value, SD, effective sample size, and correlation between outcomes) describing the main cost outcomes for which data were collected in each patient cost survey, including direct medical costs, direct non-medical costs, and indirect costs, from the WHO Global TB Programme survey database. Each of these cost outcomes was stratified by a binary tuberculosis drug resistance variable (rifampicin-resistant tuberculosis *vs* rifampicin-susceptible tuberculosis), as well as by a five-level household income quintile variable (poorest, poorer, middle, richer, and richest) representing relative differences in income within each survey sample. This income quintile variable was created by stratifying the surveyed households into five equal-sized groups according to the distribution of household income recorded in the survey sample for each country. We also obtained summary statistics for patient household income, stratified by income quintile. As tuberculosis-affected households are on average poorer than the general population,^15^ these quintile definitions will not reflect the income distribution of tuberculosis-affected households with respect to the general population.

Patient cost survey data were inflated to 2021 values in local currency using the average country consumer price index,^16^ and converted to 2021 US dollars using period-average market exchange rates.^17^

### Model specification

We constructed prediction models for three cost outcomes reported in the patient cost surveys: direct medical costs, direct non-medical costs, and indirect costs. For each of these cost outcomes we constructed models for the arithmetic mean cost for patients with tuberculosis in each country, specified as generalised linear regression models, assuming a Gamma distributed outcome and a log link function. Using the same regression approach (Gamma generalised linear regression models with log link function), we also constructed a prediction model for the arithmetic mean of annual household income for patients with tuberculosis reported in the patient cost surveys. Estimated costs for the three cost outcomes (direct medical, direct non-medical, and indirect) were summed to calculate the overall total cost per tuberculosis disease episode.

We compiled data on covariates potentially associated with the different outcomes (the three cost outcomes as well as household income): logged gross domestic product (GDP) per capita;^17^ health expenditure as a percentage of GDP;^17^ the tuberculosis incidence;^13^ the percentage of new patients with rifampicin-resistant tuberculosis;^13^ and tuberculosis treatment coverage (approximated as the number of officially reported new cases of tuberculosis divided by the estimated incidence, for a given year).^13^ In regressions using data stratified by income quintile we also included indicator variables for income quintile. In regressions using data stratified by drug resistance status we included an indicator variable for rifampicin-susceptible tuberculosis. For each regression model we identified the combination of covariates with the best predictive performance, operationalised as the set of covariates that minimised Akaike's Information Criterion for the fitted model (implemented using the ‘bestglm’ R package, version 0.37.3).^18^ All analyses were conducted in R (version 4.1.3).^19^

### Predicted outcomes

We compiled data on model covariates, described previously, for all 135 LMICs. To reduce potential errors associated with out-of-sample extrapolation we truncated covariate values to the ranges represented by the countries included in the patient cost study sample. Using the fitted regression models, we predicted cost outcomes (direct medical costs, direct non-medical costs, and indirect costs) for each LMIC in 2021, stratified by drug-resistance stratum and income quintile. We also predicted household income stratified by income quintile. To report mean per-episode cost outcomes for each country (ie, not stratified by income quintile or drug resistance), we averaged the predicted cost outcomes across income quintiles. To calculate total costs at the country level, we multiplied per-episode patient costs for a given stratum (income quintile or drug-resistance status) by the number of individuals treated for tuberculosis within that stratum.^1^ The total number of individuals treated for tuberculosis was based on country-reported notifications data for 2021,^1^ and we divided this value by 5 to obtain the total number of treated individuals within each income quintile. To calculate the total number of treated individuals within each drug-resistance stratum, we multiplied total notifications by the fraction of incident cases with rifampicin-resistant tuberculosis and rifampicin-susceptible tuberculosis, respectively, based on WHO epidemiological estimates.^1^ We assessed in-sample fit by comparing observed versus predicted values for the sample of cost surveys.

### Proportion of households experiencing catastrophic costs

We defined catastrophic total costs as patient costs incurred during an episode of tuberculosis disease that exceeded 20% of the patient's total annual household income, according to WHO guidance on the threshold to be considered catastrophic for a household's ability to pay for basic subsistence needs,^12^ and assuming that each tuberculosis case originated from a unique household (based on the low absolute prevalence of tuberculosis in households of individuals diagnosed with tuberculosis).^20^ This outcome requires comparison of household income with total costs faced by patients with tuberculosis at the household level, and therefore depends on the joint distribution of tuberculosis costs and household income. We simulated these distributions based on the results of the prediction models and other study data: first, for each country and income quintile, we simulated the three cost outcomes (direct medical costs, direct non-medical costs, and indirect costs) and household income for a hypothetical cohort of 10 000 patients. We assumed that the distribution of these patient-level values followed a Log-Normal density function (as these values were non-negative and right-skewed), with a mean value based on the results of the prediction models, and a coefficient of variation (SD divided by mean) calculated directly from the study data (averaged across the 22 survey samples). We estimated correlations between the cost and income outcomes from the empirical data (averaged across the 22 survey samples) and modelled this dependence structure in the simulated data using a Gaussian copula (a multivariate cumulative distribution function defined on the unit interval, used to induce correlations between random variables). Using this approach, we simulated correlations between cost and income outcomes by matching the correlation estimates from the empirical data. Second, we summed direct medical, direct non-medical, and indirect costs to calculate the total cost per patient.^12^ Finally, we assessed whether these total costs met the criterion for catastrophic total costs (ie, greater than 20% of annual household income) for each simulated household and averaged across all 10 000 simulated households to calculate the proportion of households facing catastrophic total costs. This sequence of calculations was performed separately for each country and income quintile, matching the mean cost generated by the prediction models.

We validated this approach by comparing modelled results with the empirical estimate for the fraction of patients facing catastrophic total costs in each study sample. We also considered an alternative definition of patient costs that only included the direct medical costs. By excluding direct non-medical and indirect costs, this definition will produce a conservative estimate of the fraction of patients facing catastrophic total costs.

### Uncertainty analysis

We estimated equal-tailed 95% uncertainty intervals for each study outcome, representing parameter uncertainty in the fitted regression models. To do so, we simulated 1000 values from the regression coefficients for each prediction model, using the coefficient point estimates and a variance-covariance matrix generated from the fitted regression models. We used these coefficient sets to generate 1000 values for each study outcome, and calculated intervals as the 2·5th and 97·5th percentiles of the distribution of these results.

### Role of the funding source

Employees of the funder participated as study investigators.

## Results

Using data from 22 national surveys of costs faced by patients with tuberculosis and their households, predictive models were fitted for each of the three patient cost outcomes and the patient household income outcome. Table 2 presents the final prediction model for each outcome. Coefficient estimates from these fitted models are provided in the appendix (pp 3–5).

**Table 2:** Best-fit prediction model for specified outcome

	**Stratification**	**Model**
Direct medical costs	Rifampicin-sensitive or rifampicin-resistant tuberculosis	rs_ind + health_ex_pct + rr_pct + c_cdr
Direct non-medical costs	Rifampicin-sensitive or rifampicin-resistant tuberculosis	rs_ind + health_ex_pct
Indirect costs	Rifampicin-sensitive or rifampicin-resistant tuberculosis	rs_ind + log_gdp_pc + health_ex_pct + c_cdr
Direct medical costs	Income quintile	quintile + health_ex_pct + tb_inc_rate + rr_pct + c_cdr
Direct non-medical costs	Income quintile	quintile + log_gdp_pc + health_ex_pct + tb_inc_rate + c_cdr
Indirect costs	Income quintile	quintile + health_ex_pct + rr_pct + c_cdr
Household income	Income quintile	quintile + log_gdp_pc + health_ex_pct + tb_inc_rate + rr_pct

rs_ind=rifampicin-sensitive tuberculosis (*vs* rifampicin-resistant tuberculosis) indicator. health_ex_pct=health expenditure as a percentage of gross domestic product. rr_pct=percentage of patients with rifampicin-resistant tuberculosis among new patients with tuberculosis. c_cdr=case detection rate (ie, tuberculosis treatment coverage). log_gdp_pc=log gross domestic product per capita. quintile=household income quintile (ie, poorest, poorer, middle, richer, and richest). tb_inc_rate=estimated tuberculosis incidence rate.

For each of the three patient cost outcomes, summary results across 135 LMICs and by WHO region per tuberculosis episode are provided in table 3. Estimated per-tuberculosis-episode direct medical costs across 135 LMICs were US$211 (95% uncertainty interval 154–302), direct non-medical costs were $512 (428–620), and indirect costs were $530 (423–663), for an overall patient cost of $1253 (1127–1417) per tuberculosis episode. Indirect costs contributed the most to total patient costs (42% [38–47%]), followed by direct non-medical costs (41% [38–44%]). The highest estimated per-episode total patient costs were in the region of the Americas, followed by the European region, Western Pacific region, Eastern Mediterranean region, African region, and South-East Asian region. Estimates for individual countries are provided in the appendix (pp 6–37).

**Table 3:** Per-episode predicted direct medical costs, direct non-medical costs, indirect costs, and overall costs, stratified by country grouping

		**Direct medical**	**Direct non-medical**	**Indirect**	**Overall**
All countries*		211 (154–302)	512 (428–620)	530 (423–663)	1253 (1127–1417)
Drug-resistance status					
	Rifampicin-susceptible tuberculosis	191 (106–342)	386 (264–535)	464 (304–692)	1042 (755–1445)
	Rifampicin-resistant tuberculosis	474 (217–910)	1683 (1146–2437)	1663 (1020–2687)	3820 (2913–5108)
WHO world region					
	African region	200 (147–282)	481 (380–621)	670 (526–846)	1352 (1224–1523)
	Region of the Americas	532 (303–897)	1850 (1221–2732)	1240 (818–1844)	3622 (2808–4702)
	Eastern Mediterranean region	256 (190–344)	500 (404–634)	649 (511–824)	1404 (1292–1548)
	European region	606 (288–1137)	1071 (779–1457)	967 (601–1557)	2644 (2109–3376)
	South-East Asian region	125 (90–170)	351 (285–426)	325 (258–407)	801 (744–875)
	Western Pacific region	337 (173–630)	691 (494–956)	663 (458–994)	1690 (1362–2124)
Income quintile					
	Poorest	172 (100–278)	364 (255–494)	120 (83–168)	656 (496–851)
	Poorer	142 (84–229)	434 (304–589)	242 (170–340)	818 (683–994)
	Middle	175 (98–292)	471 (337–664)	361 (250–499)	1007 (771–1321)
	Richer	199 (114–317)	516 (367–719)	603 (424–819)	1318 (1061–1611)
	Richest	369 (215–599)	776 (554–1052)	1322 (940–1885)	2467 (2100–2951)

Data are estimated costs (US$) per tuberculosis episode (95% uncertainty interval). Groupings are weighted by 2021 notified tuberculosis cases.

* All countries includes 135 low-income and middle-income countries analysed.

In 2021, the total direct medical costs incurred by patients treated for tuberculosis summed across all notified tuberculosis cases in 135 LMICs were estimated as $1287 million ($699–2318 million), direct non-medical costs were $2792 million ($1905–3892), and indirect costs were $3261 million ($2117–4916), for total patient costs of $7340 million ($5356–10 126; table 4). The majority of each predicted cost outcome was attributable to rifampicin-susceptible tuberculosis (90% of direct medical costs, 84% of direct non-medical costs, and 86% of indirect costs), despite the higher per-episode costs for rifampicin-resistant tuberculosis shown in table 3, given the rates of rifampicin-resistant tuberculosis prevalence across the 135 LMICs (5% on average).^13^

**Table 4:** Total direct medical costs, direct non-medical costs, and indirect costs estimated for notified tuberculosis cases in 2021, stratified by rifampicin-sensitive and rifampicin-resistant tuberculosis and region (US$ millions)

			**Direct medical**	**Direct non-medical**	**Indirect**	**Overall**
All countries*			1287 (699–2318)	2792 (1905–3892)	3261 (2117–4916)	7340 (5356–10 126)
Rifampicin-susceptible tuberculosis						
	All countries*		1161 (642–2077)	2345 (1601–3246)	2820 (1846–4203)	6327 (4583–8770)
	WHO world region					
		African region	293 (169–487)	694 (460–995)	807 (498–1266)	1794 (1342–2431)
		Region of the Americas	62 (26–135)	174 (87–313)	247 (111–506)	484 (296–792)
		Eastern Mediterranean region	103 (58–176)	201 (136–283)	212 (130–348)	516 (395–684)
		European region	32 (10–82)	41 (27–58)	56 (33–96)	129 (77–217)
		South-East Asian region	370 (201–644)	825 (534–1210)	899 (546–1424)	2094 (1563–2795)
		Western Pacific region	302 (113–743)	410 (281–562)	598 (321–1074)	1310 (790–2137)
Rifampicin-resistant tuberculosis						
	All countries*		126 (58–241)	446 (304–647)	441 (271–713)	1013 (773–1355)
	WHO world region					
		African region	22 (12–41)	104 (63–164)	108 (60–180)	234 (178–306)
		Region of the Americas	5 (2–10)	25 (14–46)	28 (13–54)	58 (43–81)
		Eastern Mediterranean region	8 (4–14)	31 (19–50)	25 (13–45)	64 (46–87)
		European region	40 (10–103)	104 (69–154)	110 (56–207)	254 (181–382)
		South-East Asian region	27 (14–47)	119 (77–174)	99 (62–154)	245 (180–319)
		Western Pacific region	24 (8–59)	63 (44–90)	71 (37–130)	158 (113–237)

Data are total costs in US$ millions (95% uncertainty intervals).

* All countries includes 135 low-income and middle-income countries analysed.

For the proportions of households with tuberculosis experiencing catastrophic total costs, summary results across 135 LMICs and by WHO region are provided in table 5. Estimates for individual countries are provided in the appendix (pp 38–53). The estimated proportions of tuberculosis-affected households experiencing catastrophic total costs decreased with increasing wealth: 75·2% (95% uncertainty interval 70·3–80·0) in the poorest quintile; 59·8% (51·8–67·8) in the poorer quintile; 51·2% (42·7–60·2) in the middle quintile; 45·9% (36·1–56·4) in the richer quintile; and 42·5% (34·3–51·5) in the richest quintile. Overall, 54·9% (47·0–63·2) of tuberculosis-affected households were estimated to have experienced total costs that were catastrophic in 2021.

**Table 5:** Predicted proportion of households experiencing catastrophic total costs due to tuberculosis, stratified by income quintile and region, weighted by tuberculosis incidence in 2020

		**Poorest**	**Poorer**	**Middle**	**Richer**	**Richest**	**Overall**
All countries*		75·2% (70·3–80·0)	59·8% (51·8–67·8)	51·2% (42·7–60·2)	45·9% (36·1–56·4)	42·5% (34·3–51·5)	54·9% (47·0–63·2)
WHO world region							
	African region	83·1% (78·2–87·5)	73·2% (64·9–80·6)	65·7% (56·3–74·8)	60·9% (49·8–71·4)	54·5% (44·9–64·0)	67·5% (58·8–75·7)
	Region of the Americas	82·4% (78·0–86·5)	71·0% (63·0–79·0)	63·0% (53·8–72·4)	56·7% (45·7–68·1)	50·5% (41·5–60·5)	64·7% (56·4–73·3)
	Eastern Mediterranean region	80·8% (76·5–84·7)	68·7% (62·0–75·2)	60·5% (52·5–68·3)	55·4% (45·8–64·7)	50·5% (42·8–59·4)	63·2% (55·9–70·5)
	European region	72·0% (65·1–78·4)	54·6% (44·6–65·3)	45·7% (35·6–56·8)	40·1% (29·8–52·0)	37·9% (29·5–47·8)	50·1% (40·9–60·1)
	South-East Asian region	71·8% (67·8–76·0)	53·6% (46·5–61·0)	44·3% (36·8–52·6)	38·6% (29·8–48·5)	36·6% (29·1–45·0)	49·0% (42·0–56·6)
	Western Pacific region	69·1% (61·6–76·3)	50·7% (40·5–61·4)	42·0% (32·4–52·8)	37·2% (26·8–49·1)	35·5% (27·2–45·1)	46·9% (37·7–56·9)

Data are % (95% uncertainty level).

* All countries includes 135 low-income and middle-income countries analysed.

We assessed in-sample fit by comparing observed versus predicted values (appendix p 54) and residual versus model fits (appendix p 55) for the sample of cost surveys. We present six subplots: (A) direct medical costs for rifampicin-susceptible tuberculosis; (B) direct medical costs for rifampicin-resistant tuberculosis; (C) direct non-medical costs for rifampicin-susceptible tuberculosis; (D) direct non-medical costs for rifampicin-resistant tuberculosis; (E) indirect costs for rifampicin-susceptible tuberculosis; and (F) indirect costs for rifampicin-resistant tuberculosis in the appendix (p 54). These plots indicated modelled costs were closer to empirical costs for rifampicin-susceptible tuberculosis costs than rifampicin-resistant tuberculosis costs. Consistent with this, empirical rifampicin-resistant tuberculosis costs had greater heterogeneity across countries, with the shape parameter of the Gamma generalised linear model ranging from 1·12 to 1·90.

We report results (stratified by drug resistance category) for a subset of six example countries (ie, Democratic Republic of the Congo, Kenya, Mali, Thailand, Tanzania, and Uganda) in the appendix (pp 56–57), selected to represent a range of characteristics including tuberculosis burden, WHO region, and World Bank income level. Each of these countries has also conducted a patient cost survey, allowing comparison of empirical and modelled estimates. The modelled cost estimates varied most widely for indirect compared with direct costs, as indirect costs had the greatest heterogeneity at the country level.

We report results for the six example countries stratified by income quintile in the appendix (pp 58–59). In most instances, the predicted proportions of tuberculosis-affected households facing catastrophic total costs were similar to the observed proportions. Predicted proportions were more likely to differ from observed proportions in countries in which the observed proportions differed substantially from LMIC and regional means (table 5).

## Discussion

In this study we synthesised data from 22 nationally representative surveys to estimate the costs faced by patients with tuberculosis and their households, and the risks of catastrophic total costs for tuberculosis-affected households, in 135 LMICs. For 2021, the mean patient cost across all 135 LMICs was estimated to be $1253 (1127–1417) per tuberculosis episode. This mean cost per tuberculosis episode represents nearly 30% of annual per capita GDP in these countries, and is substantially higher than previously estimated for LMICs.^2^ Overall, we estimated that 54·9% (47·0–63·2) of tuberculosis-affected households experienced catastrophic total costs in 2021. Although the estimates varied widely among individual countries, the comparison with empirical patient cost survey data showed that modelled costs were all within the observed range of empirical costs.

Evidence on costs incurred by patients with tuberculosis can be used to inform approaches that will reduce these costs to affected households, such as improving access to care through service integration and decentralisation, expanding and adjusting health financing schemes, expanding free tuberculosis care policies, improving tuberculosis-specific social support, and enhancing social protection schemes.^9^ Collection of patient cost data can also provide evidence on the impact of efforts to reduce these costs.^21^ The results of this analysis can support country-level decision making regarding the health sector and multisectoral policy responses in countries that have yet to conduct a national patient cost survey.^22^, ^23^ Enacting policy responses in these areas also contributes to reaching the Sustainable Development Goals 1 and 3 to end poverty in all its forms everywhere and to achieve universal health coverage, including financial risk protection for all, respectively.^24^

In the meta-regression analysis, patient costs were predicted to be higher for rifampicin-resistant tuberculosis compared with rifampicin-susceptible tuberculosis, which is in line with current empirical data of costs for patients with tuberculosis. However, WHO recently recommended a shorter duration for rifampicin-resistant tuberculosis treatment regimens,^25^ which could positively impact tuberculosis-affected households by reducing patient costs as these new regimens are introduced. An increasing trend in country-specific patient costs was estimated with increasing per-capita GDP (a proxy for country health system costs) and health expenditure as a percentage of GDP. The regression covariate results can provide some information about country-level cost differences according to the resource context, current tuberculosis treatment coverage, and prevalence of rifampicin-resistant tuberculosis, but further research is needed to identify modifiable features of tuberculosis programmes that can also mitigate patient costs.

Our study has several limitations. First, the 22 countries included in the dataset were not a representative sample of all LMICs. The countries represented by the sampled studies were generally of lower GDP per capita (average $2820 *vs* $4310) and had a lower percentage of patients with rifampicin-resistant tuberculosis among new patients with tuberculosis (average 0·4% *vs* 2·8%) compared with the 135 LMICs for which we report modelled estimates. To reduce potential errors associated with out-of-sample extrapolation we truncated covariate values to the ranges represented by the countries included in the patient cost study sample, yet the robustness of these estimates will be lower for countries that differ from those included in the dataset. Second, it is possible that the meta-regression approach omitted important individual-level or country-level predictors of per-patient costs. The large residual variance of the meta-regression models suggests that there are additional factors determining patient costs, either systemic or unique to individual countries. For this reason, the estimates produced by this analysis should not be considered equivalent to empirical data collected within a country of interest. Moreover, as we only had access to group-level data from each survey, we were not able to investigate individual-level cost determinants beyond income quintile and drug resistance status, yet these factors could probably explain individual-level variation in patient costs.^26^, ^27^ Additional research on the determinants of patient costs—both within and between countries—is needed to inform efforts to address these costs. Third, the survey data that this study relied on only included patients at national tuberculosis programme facilities; therefore, generalisability might be reduced. The results of this analysis provide no evidence about the costs faced by people with tuberculosis who are treated in facilities not linked to the national tuberculosis programme (and not officially reported), or who remain undiagnosed and untreated. These two groups of individuals could represent a considerable fraction of all individuals developing tuberculosis disease in a given year (about 40% globally, including both unreported and undiagnosed or untreated individuals, in 2020 and 2021), especially in countries with a large private sector and those with poorer overall health service coverage.^1^ They might receive one or more episodes of non-curative treatment, and might experience an extended period of morbidity and reduced productivity, as well as higher mortality rates. As these individuals are excluded from the analysis, the estimates of total costs could be a conservative estimate of the full household economic burden of tuberculosis. Fourth, there were differences between surveys in the variable describing household ability-to-pay, with 18 surveys recording self-reported income and four recording self-reported household expenditure (table 1). This variation requires additional assumptions when pooling the data compared with a situation in which all surveys report an ability-to-pay in the same manner. Moreover, both approaches have noted deficiencies as measures of household ability to absorb expenditure shocks.^28^ Additionally, we only included one definition of the catastrophic cost threshold (>20%), per the current definition used by WHO's Global TB Programme; higher (or lower) thresholds would lead to lower (or higher) estimates of the number of households experiencing catastrophic costs.^1^

Across LMICs, patients with tuberculosis and their households face substantial economic losses associated with diagnosis and treatment, and the WHO End TB Strategy target that no tuberculosis-affected household faces catastrophic total costs has probably not been achieved in any LMIC. Actions to mitigate and eliminate these cost barriers are required in all LMICs. The estimates reported in this study could be useful to inform these actions in countries that have yet to conduct a national survey.

## Data sharing

Analytic code and study inputs based on publicly available databases are available online. Summary statistics of country tuberculosis patient cost surveys are published in the WHO Global TB Reports.

## Declaration of interests

TY, PN, NN, KF, and IGB are employees of WHO. AP and NAM declare no competing interests.
