# Supplementary material for: Costs incurred by people receiving tuberculosis treatment in low-income and middle-income countries: a meta-regression analysis
Source: Lancet Glob Health. 2023 Sep 19;11(10):e1640–7. doi: 10.1016/S2214-109X(23)00369-8 (PMC10522775; doi:10.1016/S2214-109X(23)00369-8)
Supplement: Supplementary appendix [file mmc1.pdf]

THE LANCET
Global Health

Supplementary appendix

This appendix formed part of the original submission and has been peer reviewed. We post it as supplied by the authors.

Supplement to: Portnoy A, Yamanaka T, Nguhiu P, et al. Costs incurred by people receiving tuberculosis treatment in low-income and middle-income countries: a meta-regression analysis. *Lancet Glob Health* 2023; **11:** e1640–47.

Supplementary Material for "Costs incurred by people receiving TB treatment in low- and middle-income countries: a meta-regression analysis"

Table of Contents

Figure S1. Overview of the tuberculosis patient cost survey cross-sectional design and analytical approach. 2

Table S1. Results for regressions of RS-/RR-TB direct medical costs on predictors 3

Table S2. Results for regressions of RS-/RR-TB direct non-medical costs on predictors 3

Table S3. Results for regressions of RS-/RR-TB indirect costs on predictors 3

Table S4. Results for regressions of TB direct medical costs by quintile on predictors 4

Table S5. Results for regressions of TB direct non-medical costs by quintile on predictors 4

Table S6. Results for regressions of TB indirect costs by quintile on predictors 5

Table S7. Results for regressions of annual household income by quintile on predictors 5

Table S8. Predicted direct medical unit cost for notified tuberculosis (TB) cases in 2021, stratified by rifampicin-sensitive (RS) and rifampicin-resistant (RR) by country. 6

Table S9. Predicted direct non-medical unit cost for notified tuberculosis (TB) cases in 2021, stratified by rifampicin-sensitive (RS) and rifampicin-resistant (RR) by country. 14

Table S10. Predicted indirect unit cost for notified tuberculosis (TB) cases in 2021, stratified by rifampicin-sensitive (RS) and rifampicin-resistant (RR) by country. 22

Table S11. Predicted direct medical, direct non-medical, and indirect unit cost overall for notified tuberculosis (TB) cases in 2021. 30

Table S12. Predicted proportion of households experiencing catastrophic costs due to tuberculosis, stratified by income quintile*. 38

Table S13. Predicted proportion of households experiencing catastrophic direct medical costs due to tuberculosis, stratified by income quintile*. 46

Figure S14. Comparison of predicted costs and World Health Organization Tuberculosis Patient Cost Survey (TB-PCS) data for direct medical costs (DM), direct non-medical costs (NM), and indirect costs (IND), stratified by rifampicin-sensitive (RS) and rifampicin-resistant (RR) TB. 54

Figure S15. Comparison of residuals vs. model fit for direct medical costs (DM), direct non-medical costs (NM), and indirect costs (IND). 55

Table S16. Comparison of World Health Organization Tuberculosis Patient Cost Survey (TB-PCS) data to predicted patient costs by country and drug resistance category (RR = rifampicin-resistant; RS = rifampicin-sensitive) in 2021 USD. 56

Table S17. Comparison of World Health Organization Tuberculosis Patient Cost Survey (TB-PCS) data to predicted proportions of households experiencing catastrophic costs by country and income quintile. 58

Figure S1. Overview of the tuberculosis patient cost survey cross-sectional design and analytical approach.

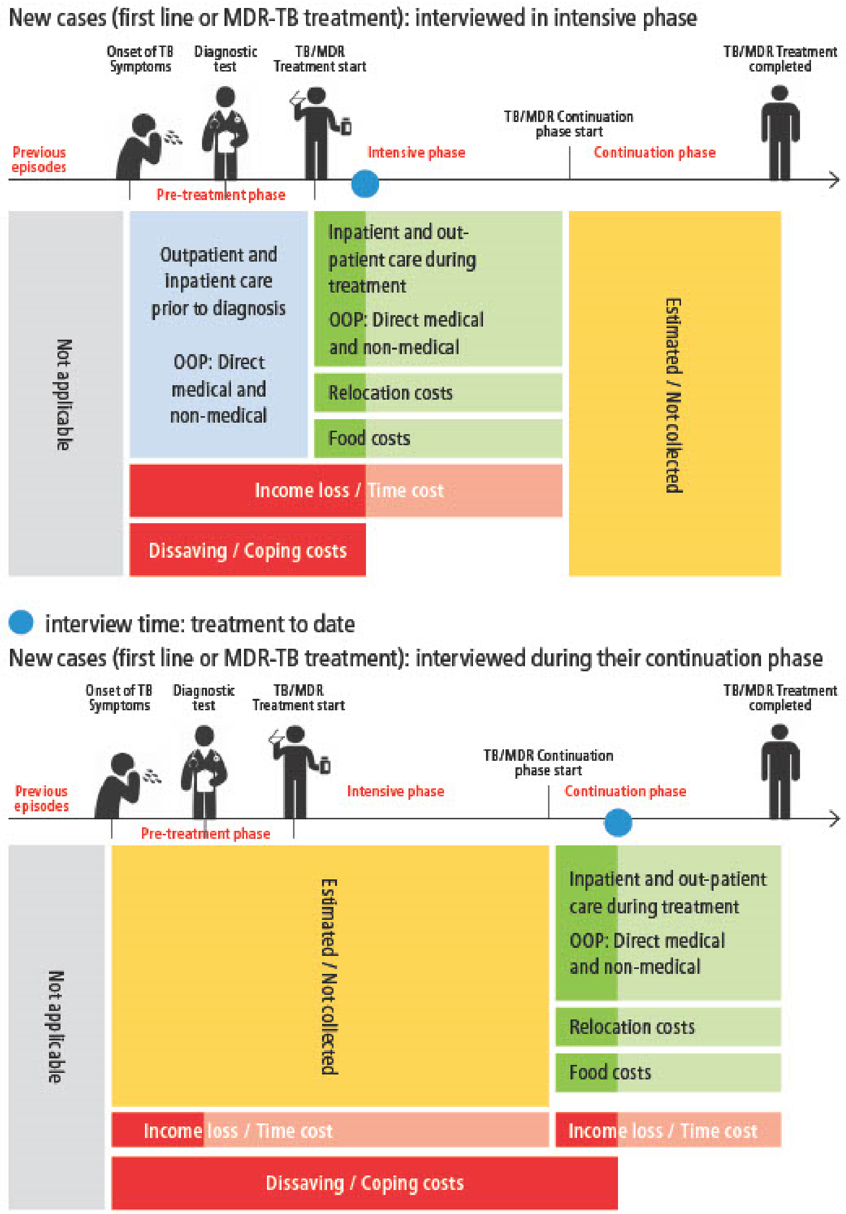

Note: This figure has been re-purposed from the World Health Organization's "Tuberculosis patient cost surveys: a hand book" (https://www.who.int/publications/i/item/9789241513524). The blue dot indicates the interview moment. Darker shades of blue, green, and red represent retrospective data collected at the interview. Lighter shades of green and red represent extrapolation of costs into the future. Yellow means costs are estimated based on both information from the interviewed person and imputations based on data from other patients' data. OOP refers to out-of-pocket payments.

Table S1. Results for regressions of RS-/RR-TB direct medical costs on predictors

Variable	Mean coefficient (standard error)
Intercept	5·291 (0·915)
rs_ind	-0·645 (0·339)
health_ex_pct	17·654 (9·206)
rr_pct	17·191 (11·283)
c_cdr	-1·493 (1·249)

Note: c_cdr = case detection rate (i.e., tuberculosis (TB) treatment coverage); rs_ind = rifampicin-sensitive TB (vs. rifampicin-resistant (RR) TB) indicator; health_ex_pct = health expenditure as a percentage of gross domestic product; rr_pct = percentage of patients with RR-TB among new TB patients.

S1 Model Equation, where i = country:

$$\hat{c}_i = \exp\,(\beta_0 + \beta_1 * rs\_ind + \beta_2 * health\_ex\_pct_i + \beta_3 * rr\_pct_i + \beta_4 * c\_cdr_i)$$

Table S2. Results for regressions of RS-/RR-TB direct non-medical costs on predictors

Variable	Mean coefficient (standard error)
Intercept	6·319 (0·351)
rs_ind	-1·350 (0·249)
health_ex_pct	21·398 (6·469)

Note: rs_ind = rifampicin-sensitive tuberculosis (TB) (vs. rifampicin-resistant (RR) TB) indicator; health_ex_pct = health expenditure as a percentage of gross domestic product

S2 Model Equation, where i = country:

$$\hat{c}_i = \exp\,(\beta_0 + \beta_1 * rs\_ind + \beta_2 * health\_ex\_pct_i)$$

Table S3. Results for regressions of RS-/RR-TB indirect costs on predictors

Variable	Mean coefficient (standard error)
Intercept	5·117 (1·437)
rs_ind	-1·093 (0·269)
log_gdp_pc	0·237 (0·176)
health_ex_pct	24·119 (7·403)
c_cdr	-1·501 (0·900)

Note: c_cdr = case detection rate (i.e., tuberculosis (TB) treatment coverage); rs_ind = rifampicin-sensitive TB (vs. rifampicin-resistant (RR) TB) indicator; health_ex_pct = health expenditure as a percentage of gross domestic product (GDP); log_gdp_pc = log GDP per capita.

S3 Model Equation, where i = country:

$$\hat{c}_i = \exp\,(\beta_0 + \beta_1 * rs\_ind \; + \beta_2 * log\_gdp\_pc_i + \beta_3 * health\_ex\_pct_i + \beta_4 * c\_cdr_i)$$

Table S4. Results for regressions of TB direct medical costs by quintile on predictors

Variable	Mean coefficient (standard error)
Intercept	5·107 (0·716)
quintile2	-0·185 (0·327)
quintile3	0·023 (0·327)
quintile4	0·157 (0·327)
quintile5	0·766 (0·327)
health_ex_pct	23·196 (6·507)
tb_inc_rate	-212·697 (87·963)
rr_pct	19·437 (6·698)
c_cdr	-2·268 (0·813)

Note: c_cdr = case detection rate (i.e., tuberculosis (TB) treatment coverage); health_ex_pct = health expenditure as a percentage of gross domestic product; quintile = household income quintile (poorest/poorer/middle/richer/richest); rr_pct = percentage of patients with rifampicin-resistant (RR) TB among new TB patients; tb_inc_rate = estimated TB incidence rate.

S4 Model Equation, where i = country:

$$\hat{c}_i = \exp\left(\beta_0 + \beta_1 * quintile2 + \beta_2 * quintile3 + \beta_3 * quintile4 + \beta_4 * quintile5 + \beta_5 * health\_ex\_pct_i + \beta_6 * tb\_inc\_rate_i + \beta_7 * rr\_pct_i + \beta_8 * c\_cdr_i\right)$$

Table S5. Results for regressions of TB direct non-medical costs by quintile on predictors

Variable	Mean coefficient (standard error)
Intercept	4·327 (0·812)
quintile2	0·178 (0·227)
quintile3	0·257 (0·227)
quintile4	0·352 (0·227)
quintile5	0·761 (0·227)
log_gdp_pc	0·230 (0·096)
health_ex_pct	22·686 (4·483)
tb_inc_rate	-216·397 (61·368)
c_cdr	-1·407 (0·504)

Note: c_cdr = case detection rate (i.e., tuberculosis (TB) treatment coverage); health_ex_pct = health expenditure as a percentage of gross domestic product (GDP); log_gdp_pc = log GDP per capita; quintile = household income quintile (poorest/poorer/middle/richer/richest); tb_inc_rate = estimated TB incidence rate.

S5 Model Equation, where i = country:

$$\hat{c}_i = \exp\left(\beta_0 + \beta_1 * quintile2 + \beta_2 * quintile3 + \beta_3 * quintile4 + \beta_4 * quintile5 + \beta_5 * log\_gdp\_pc_i + \beta_6 * health\_ex\_pct_i + \beta_7 * tb\_inc\_rate_i + \beta_8 * c\_cdr_i\right)$$

Table S6. Results for regressions of TB indirect costs by quintile on predictors

Variable	Mean coefficient (standard error)
Intercept	3·984 (0·422)
quintile2	0·714 (0·230)
quintile3	1·107 (0·230)
quintile4	1·620 (0·230)
quintile5	2·403 (0·230)
health_ex_pct	27·496 (4·220)
rr_pct	10·999 (4·673)
c_cdr	-1·486 (0·555)

Note: c_cdr = case detection rate (i.e., tuberculosis (TB) treatment coverage); health_ex_pct = health expenditure as a percentage of gross domestic product; quintile = household income quintile (poorest/poorer/middle/richer/richest); rr_pct = percentage of patients with rifampicin-resistant (RR) TB among new TB patients.

S6 Model Equation, where i = country:

$$\hat{c}_i = \exp\,(\beta_0 + \beta_1 * quintile2 + \beta_2 * quintile3 + \beta_3 * quintile4 + \beta_4 * quintile5 + \beta_5 * health\_ex\_pct_i + \beta_6 * rr\_pct_i + \beta_7 * c\_cdr_i)$$

Table S7. Results for regressions of annual household income by quintile on predictors

Variable	Mean coefficient (standard error)
Intercept	3·399 (0·392)
quintile2	0·763 (0·118)
quintile3	1·209 (0·118)
quintile4	1·642 (0·118)
quintile5	2·481 (0·118)
log_gdp_pc	0·377 (0·050)
health_ex_pct	4·613 (2·370)
tb_inc_rate	-145·532 (31·334)
rr_pct	12·285 (2·170)

Note: health_ex_pct = health expenditure as a percentage of gross domestic product (GDP); log_gdp_pc = log GDP per capita; quintile = household income quintile (poorest/poorer/middle/richer/richest); rr_pct = percentage of patients with rifampicin-resistant (RR) tuberculosis (TB) among new TB patients; tb_inc_rate = estimated TB incidence rate. Outcome represents self-reported annual income.

S7 Model Equation, where i = country:

$$\hat{c}_i = \exp\,(\beta_0 + \beta_1 * quintile2 + \beta_2 * quintile3 + \beta_3 * quintile4 + \beta_4 * quintile5 + \beta_5 * log\_gdp\_pc_i + \beta_6 * health\_ex\_pct_i + \beta_7 * tb\_inc\_rate_i + \beta_8 * rr\_pct_i)$$

Table S8. Predicted direct medical unit cost for notified tuberculosis (TB) cases in 2021, stratified by rifampicin-sensitive (RS) and rifampicin-resistant (RR) by country.

Country	Code	Region	Income level	Direct medical costs	
				RS	RR
Afghanistan	AFG	EMR	LIC	510 (156–1309)	979 (283–2489)
Angola	AGO	AFR	LMIC	158 (82–284)	302 (152–534)
Albania	ALB	EUR	UMIC	146 (76–247)	281 (147–472)
Argentina	ARG	AMR	UMIC	979 (194–3206)	1870 (351–5815)
Armenia	ARM	EUR	UMIC	1093 (219–3415)	2086 (412–6485)
American Samoa	ASM	WPR	UMIC	216 (51–605)	417 (94–1112)
Azerbaijan	AZE	EUR	UMIC	333 (90–954)	634 (175–1727)
Burundi	BDI	AFR	LIC	356 (154–733)	683 (275–1405)
Benin	BEN	AFR	LMIC	135 (58–286)	259 (111–500)
Burkina Faso	BFA	AFR	LIC	154 (91–248)	295 (168–470)
Bangladesh	BGD	SEAR	LMIC	73 (33–147)	141 (59–264)
Bulgaria	BGR	EUR	UMIC	370 (133–829)	708 (246–1498)
Bosnia and Herzegovina	BIH	EUR	UMIC	347 (68–1076)	667 (119–1993)
Belarus	BLR	EUR	UMIC	468 (136–1253)	892 (237–2226)
Belize	BLZ	AMR	UMIC	113 (52–215)	219 (97–418)
Bolivia	BOL	AMR	LMIC	265 (115–538)	509 (210–1001)
Brazil	BRA	AMR	UMIC	333 (103–807)	641 (185–1616)

Country	Code	Region	Income level	Direct medical costs	
				RS	RR
Bhutan	BTN	SEAR	LMIC	299 (69–912)	569 (126–1709)
Botswana	BWA	AFR	UMIC	249 (119–464)	477 (223–891)
Central African Republic	CAF	AFR	LIC	295 (96–719)	566 (172–1309)
China	CHN	WPR	UMIC	344 (80–1034)	656 (146–1850)
Côte d'Ivoire	CIV	AFR	LMIC	309 (85–883)	587 (159–1616)
Cameroon	CMR	AFR	LMIC	141 (71–250)	272 (135–497)
Congo, Democratic Republic of the	COD	AFR	LIC	119 (66–202)	229 (121–386)
Congo	COG	AFR	LMIC	117 (56–225)	224 (108–417)
Colombia	COL	AMR	UMIC	231 (96–497)	444 (174–917)
Comoros	COM	AFR	LMIC	206 (47–645)	394 (90–1138)
Cabo Verde	CPV	AFR	LMIC	97 (44–183)	186 (85–346)
Costa Rica	CRI	AMR	UMIC	309 (59–981)	593 (100–1826)
Cuba	CUB	AMR	UMIC	272 (76–685)	524 (141–1319)
Djibouti	DJI	EMR	LMIC	84 (32–196)	162 (58–346)
Dominica	DMA	AMR	UMIC	206 (47–645)	394 (90–1138)
Dominican Republic	DOM	AMR	UMIC	420 (151–988)	803 (271–1751)
Algeria	DZA	AFR	LMIC	174 (98–281)	334 (186–557)
Ecuador	ECU	AMR	UMIC	391 (158–828)	749 (302–1572)

Country	Code	Region	Income level	Direct medical costs	
				RS	RR
Egypt	EGY	EMR	LMIC	128 (66–224)	246 (125–413)
Eritrea	ERI	AFR	LIC	165 (98–273)	317 (187–515)
Ethiopia	ETH	AFR	LIC	89 (43–167)	170 (78–307)
Fiji	FJI	WPR	UMIC	76 (32–149)	147 (61–281)
Micronesia	FSM	WPR	LMIC	216 (51–605)	417 (94–1112)
Gabon	GAB	AFR	UMIC	182 (92–341)	348 (178–612)
Georgia	GEO	EUR	UMIC	577 (160–1574)	1101 (284–2798)
Ghana	GHA	AFR	LMIC	182 (67–415)	349 (127–785)
Guinea	GIN	AFR	LIC	238 (85–588)	455 (157–1080)
Gambia	GMB	AFR	LIC	246 (88–596)	470 (167–1087)
Guinea-Bissau	GNB	AFR	LIC	681 (219–1658)	1300 (390–3069)
Equatorial Guinea	GNQ	AFR	UMIC	413 (129–1125)	786 (229–1978)
Grenada	GRD	AMR	UMIC	185 (50–523)	355 (91–945)
Guatemala	GTM	AMR	UMIC	183 (95–322)	352 (177–604)
Guyana	GUY	AMR	UMIC	133 (68–230)	255 (129–433)
Honduras	HND	AMR	LMIC	425 (157–969)	813 (280–1729)
Haiti	HTI	AMR	LMIC	182 (109–284)	349 (208–554)
Indonesia	IDN	SEAR	LMIC	127 (64–234)	244 (120–447)
India	IND	SEAR	LMIC	137 (67–258)	262 (129–470)

Country	Code	Region	Income level	Direct medical costs	
				RS	RR
Iran	IRN	EMR	LMIC	180 (66–410)	346 (125–722)
Iraq	IRQ	EMR	UMIC	408 (135–1096)	777 (231–1986)
Jamaica	JAM	AMR	UMIC	96 (37–195)	185 (71–388)
Jordan	JOR	EMR	UMIC	304 (115–669)	583 (213–1237)
Kazakhstan	KAZ	EUR	UMIC	252 (53–826)	478 (91–1477)
Kenya	KEN	AFR	LMIC	176 (97–292)	338 (185–568)
Kyrgyz Republic	KGZ	EUR	LMIC	333 (90–954)	634 (175–1727)
Cambodia	KHM	WPR	LMIC	201 (99–375)	386 (182–708)
Kiribati	KIR	WPR	LMIC	547 (143–1516)	1049 (275–2983)
Lao People’s Democratic Republic	LAO	WPR	LMIC	77 (36–143)	147 (61–273)
Lebanon	LBN	EMR	UMIC	205 (64–490)	395 (120–940)
Liberia	LBR	AFR	LIC	305 (89–789)	584 (157–1450)
Libya	LBY	EMR	UMIC	255 (119–484)	489 (220–931)
St. Lucia	LCA	AMR	UMIC	67 (27–141)	130 (49–262)
Sri Lanka	LKA	SEAR	LMIC	108 (42–224)	207 (80–433)
Lesotho	LSO	AFR	LMIC	835 (168–2543)	1594 (275–4765)
Morocco	MAR	EMR	LMIC	185 (68–405)	355 (128–793)
Moldova, Republic of	MDA	EUR	UMIC	486 (145–1299)	926 (260–2256)

Country	Code	Region	Income level	Direct medical costs	
				RS	RR
Madagascar	MDG	AFR	LIC	107 (47–204)	205 (91–391)
Maldives	MDV	SEAR	UMIC	258 (86–617)	496 (157–1110)
Mexico	MEX	AMR	UMIC	159 (93–253)	306 (174–478)
Marshall Islands	MHL	WPR	UMIC	365 (65–1199)	701 (129–2128)
Macedonia, North	MKD	EUR	UMIC	187 (75–399)	360 (142–706)
Mali	MLI	AFR	LIC	158 (82–293)	303 (155–531)
Myanmar	MMR	SEAR	LMIC	196 (107–331)	377 (214–641)
Montenegro	MNE	EUR	UMIC	290 (65–842)	557 (116–1589)
Mongolia	MNG	WPR	LMIC	461 (160–1128)	879 (297–2058)
Mozambique	MOZ	AFR	LIC	164 (63–344)	315 (118–710)
Mauritania	MRT	AFR	LMIC	80 (32–168)	154 (60–305)
Malawi	MWI	AFR	LIC	230 (110–446)	441 (196–816)
Malaysia	MYS	WPR	UMIC	82 (38–153)	158 (70–286)
Namibia	NAM	AFR	UMIC	576 (186–1422)	1102 (348–2628)
Niger	NER	AFR	LIC	288 (148–528)	551 (282–997)
Nigeria	NGA	AFR	LMIC	248 (107–523)	474 (203–969)
Nicaragua	NIC	AMR	LMIC	197 (72–428)	378 (136–813)
Nepal	NPL	SEAR	LMIC	190 (92–354)	364 (180–664)
Pakistan	PAK	EMR	LMIC	161 (86–285)	309 (165–546)

Country	Code	Region	Income level	Direct medical costs	
				RS	RR
Peru	PER	AMR	UMIC	338 (114–838)	645 (210–1453)
Philippines	PHL	WPR	LMIC	221 (118–389)	423 (222–707)
Papua New Guinea	PNG	WPR	LMIC	56 (21–124)	107 (37–226)
Korea, Democratic People's Republic of	PRK	SEAR	LIC	121 (49–244)	232 (93–463)
Paraguay	PRY	AMR	UMIC	240 (121–424)	462 (224–870)
West Bank and Gaza	PSE	EMR	LMIC	80 (32–163)	154 (62–307)
Russian Federation	RUS	EUR	UMIC	378 (77–1136)	720 (138–2064)
Rwanda	RWA	AFR	LIC	124 (59–228)	238 (116–439)
Sudan	SDN	EMR	LIC	163 (97–256)	314 (187–503)
Senegal	SEN	AFR	LMIC	93 (44–173)	179 (84–312)
Solomon Islands	SLB	WPR	LMIC	93 (40–183)	179 (76–345)
Sierra Leone	SLE	AFR	LIC	325 (121–702)	624 (230–1381)
El Salvador	SLV	AMR	LMIC	202 (87–418)	388 (161–736)
Somalia	SOM	EMR	LIC	617 (201–1546)	1175 (347–2821)
Serbia	SRB	EUR	UMIC	378 (66–1219)	724 (118–2244)
South Sudan	SSD	AFR	LIC	185 (106–300)	356 (205–585)
São Tomé and Principe	STP	AFR	LMIC	293 (116–622)	561 (213–1198)
Suriname	SUR	AMR	UMIC	1047 (215–3327)	2000 (384–6116)

Country	Code	Region	Income level	Direct medical costs	
				RS	RR
Swaziland	SWZ	AFR	LMIC	376 (160–778)	720 (302–1415)
Syrian Arab Republic	SYR	EMR	LIC	85 (39–161)	163 (72–307)
Chad	TCD	AFR	LIC	367 (109–980)	698 (204–1796)
Togo	TGO	AFR	LIC	120 (56–221)	230 (106–427)
Thailand	THA	SEAR	UMIC	113 (47–233)	218 (88–455)
Tajikistan	TJK	EUR	LMIC	643 (190–1699)	1227 (340–3032)
Turkmenistan	TKM	EUR	UMIC	144 (54–319)	278 (101–577)
Timor-Leste	TLS	SEAR	LMIC	230 (83–513)	442 (149–955)
Tonga	TON	WPR	UMIC	87 (36–173)	167 (71–326)
Tunisia	TUN	EMR	LMIC	180 (78–360)	347 (148–659)
Turkey	TUR	EUR	UMIC	119 (66–200)	228 (123–382)
Tuvalu	TUV	WPR	UMIC	255 (70–654)	491 (125–1269)
Tanzania, United Republic of	TZA	AFR	LMIC	100 (51–179)	192 (93–324)
Uganda	UGA	AFR	LIC	113 (63–189)	217 (115–358)
Ukraine	UKR	EUR	LMIC	617 (183–1652)	1177 (323–2915)
Uzbekistan	UZB	EUR	LMIC	512 (157–1344)	976 (287–2337)
St. Vincent and the Grenadines	VCT	AMR	UMIC	80 (32–163)	154 (62–307)
Venezuela	VEN	AMR	UMIC	109 (55–194)	210 (104–361)
Vietnam	VNM	WPR	LMIC	412 (122–1102)	786 (226–1999)

Country	Code	Region	Income level	Direct medical costs	
				RS	RR
Vanuatu	VUT	WPR	LMIC	66 (27–136)	127 (49–252)
Samoa	WSM	WPR	LMIC	96 (37–195)	185 (71–388)
Kosovo	XKX	EUR	UMIC	189 (83–385)	364 (153–688)
Yemen	YEM	EMR	LIC	129 (75–213)	249 (141–400)
South Africa	ZAF	AFR	UMIC	429 (157–1015)	824 (289–1917)
Zambia	ZMB	AFR	LIC	214 (96–426)	411 (188–799)
Zimbabwe	ZWE	AFR	LMIC	281 (109–628)	540 (197–1135)

* All countries include 135 low- and middle-income countries analyzed. Values in parentheses represent equal-tailed 95% credible intervals.

Table S9. Predicted direct non-medical unit cost for notified tuberculosis (TB) cases in 2021, stratified by rifampicin-sensitive (RS) and rifampicin-resistant (RR) by country.

Country	Code	Region	Income level	Direct non-medical costs	
				RS	RR
Afghanistan	AFG	EMR	LIC	1329 (553–2655)	5113 (2263–10189)
Angola	AGO	AFR	LMIC	280 (178–415)	1078 (687–1598)
Albania	ALB	EUR	UMIC	428 (293–589)	1644 (1133–2343)
Argentina	ARG	AMR	UMIC	1329 (553–2655)	5113 (2263–10189)
Armenia	ARM	EUR	UMIC	1329 (553–2655)	5113 (2263–10189)
American Samoa	ASM	WPR	UMIC	1329 (553–2655)	5113 (2263–10189)
Azerbaijan	AZE	EUR	UMIC	346 (232–490)	1328 (906–1891)
Burundi	BDI	AFR	LIC	833 (452–1376)	3204 (1787–5417)
Benin	BEN	AFR	LMIC	229 (129–365)	878 (503–1397)
Burkina Faso	BFA	AFR	LIC	428 (293–589)	1644 (1133–2343)
Bangladesh	BGD	SEAR	LMIC	229 (129–365)	878 (503–1397)
Bulgaria	BGR	EUR	UMIC	664 (400–1007)	2554 (1566–3977)
Bosnia and Herzegovina	BIH	EUR	UMIC	1050 (490–1903)	4038 (2002–7509)
Belarus	BLR	EUR	UMIC	532 (349–753)	2044 (1354–2999)
Belize	BLZ	AMR	UMIC	532 (349–753)	2044 (1354–2999)
Bolivia	BOL	AMR	LMIC	664 (400–1007)	2554 (1566–3977)
Brazil	BRA	AMR	UMIC	1329 (553–2655)	5113 (2263–10189)
Bhutan	BTN	SEAR	LMIC	346 (232–490)	1328 (906–1891)

Country	Code	Region	Income level	Direct non-medical costs	
				RS	RR
Botswana	BWA	AFR	UMIC	532 (349–753)	2044 (1354–2999)
Central African Republic	CAF	AFR	LIC	833 (452–1376)	3204 (1787–5417)
China	CHN	WPR	UMIC	428 (293–589)	1644 (1133–2343)
Côte d'Ivoire	CIV	AFR	LMIC	280 (178–415)	1078 (687–1598)
Cameroon	CMR	AFR	LMIC	346 (232–490)	1328 (906–1891)
Congo, Democratic Republic of the	COD	AFR	LIC	346 (232–490)	1328 (906–1891)
Congo	COG	AFR	LMIC	229 (129–365)	878 (503–1397)
Colombia	COL	AMR	UMIC	833 (452–1376)	3204 (1787–5417)
Comoros	COM	AFR	LMIC	428 (293–589)	1644 (1133–2343)
Cabo Verde	CPV	AFR	LMIC	428 (293–589)	1644 (1133–2343)
Costa Rica	CRI	AMR	UMIC	664 (400–1007)	2554 (1566–3977)
Cuba	CUB	AMR	UMIC	1329 (553–2655)	5113 (2263–10189)
Djibouti	DJI	EMR	LMIC	229 (129–365)	878 (503–1397)
Dominica	DMA	AMR	UMIC	428 (293–589)	1644 (1133–2343)
Dominican Republic	DOM	AMR	UMIC	532 (349–753)	2044 (1354–2999)
Algeria	DZA	AFR	LMIC	532 (349–753)	2044 (1354–2999)
Ecuador	ECU	AMR	UMIC	833 (452–1376)	3204 (1787–5417)
Egypt	EGY	EMR	LMIC	428 (293–589)	1644 (1133–2343)

Country	Code	Region	Income level	Direct non-medical costs	
				RS	RR
Eritrea	ERI	AFR	LIC	346 (232–490)	1328 (906–1891)
Ethiopia	ETH	AFR	LIC	280 (178–415)	1078 (687–1598)
Fiji	FJI	WPR	UMIC	346 (232–490)	1328 (906–1891)
Micronesia	FSM	WPR	LMIC	1329 (553–2655)	5113 (2263–10189)
Gabon	GAB	AFR	UMIC	280 (178–415)	1078 (687–1598)
Georgia	GEO	EUR	UMIC	664 (400–1007)	2554 (1566–3977)
Ghana	GHA	AFR	LMIC	280 (178–415)	1078 (687–1598)
Guinea	GIN	AFR	LIC	346 (232–490)	1328 (906–1891)
Gambia	GMB	AFR	LIC	346 (232–490)	1328 (906–1891)
Guinea-Bissau	GNB	AFR	LIC	833 (452–1376)	3204 (1787–5417)
Equatorial Guinea	GNQ	AFR	UMIC	280 (178–415)	1078 (687–1598)
Grenada	GRD	AMR	UMIC	428 (293–589)	1644 (1133–2343)
Guatemala	GTM	AMR	UMIC	532 (349–753)	2044 (1354–2999)
Guyana	GUY	AMR	UMIC	428 (293–589)	1644 (1133–2343)
Honduras	HND	AMR	LMIC	664 (400–1007)	2554 (1566–3977)
Haiti	HTI	AMR	LMIC	428 (293–589)	1644 (1133–2343)
Indonesia	IDN	SEAR	LMIC	280 (178–415)	1078 (687–1598)
India	IND	SEAR	LMIC	280 (178–415)	1078 (687–1598)
Iran	IRN	EMR	LMIC	664 (400–1007)	2554 (1566–3977)

Country	Code	Region	Income level	Direct non-medical costs	
				RS	RR
Iraq	IRQ	EMR	UMIC	346 (232–490)	1328 (906–1891)
Jamaica	JAM	AMR	UMIC	532 (349–753)	2044 (1354–2999)
Jordan	JOR	EMR	UMIC	833 (452–1376)	3204 (1787–5417)
Kazakhstan	KAZ	EUR	UMIC	280 (178–415)	1078 (687–1598)
Kenya	KEN	AFR	LMIC	428 (293–589)	1644 (1133–2343)
Kyrgyz Republic	KGZ	EUR	LMIC	346 (232–490)	1328 (906–1891)
Cambodia	KHM	WPR	LMIC	664 (400–1007)	2554 (1566–3977)
Kiribati	KIR	WPR	LMIC	1329 (553–2655)	5113 (2263–10189)
Lao People's Democratic Republic	LAO	WPR	LMIC	280 (178–415)	1078 (687–1598)
Lebanon	LBN	EMR	UMIC	1050 (490–1903)	4038 (2002–7509)
Liberia	LBR	AFR	LIC	833 (452–1376)	3204 (1787–5417)
Libya	LBY	EMR	UMIC	532 (349–753)	2044 (1354–2999)
St. Lucia	LCA	AMR	UMIC	346 (232–490)	1328 (906–1891)
Sri Lanka	LKA	SEAR	LMIC	346 (232–490)	1328 (906–1891)
Lesotho	LSO	AFR	LMIC	1329 (553–2655)	5113 (2263–10189)
Morocco	MAR	EMR	LMIC	428 (293–589)	1644 (1133–2343)
Moldova, Republic of	MDA	EUR	UMIC	532 (349–753)	2044 (1354–2999)
Madagascar	MDG	AFR	LIC	346 (232–490)	1328 (906–1891)

Country	Code	Region	Income level	Direct non-medical costs	
				RS	RR
Maldives	MDV	SEAR	UMIC	833 (452–1376)	3204 (1787–5417)
Mexico	MEX	AMR	UMIC	428 (293–589)	1644 (1133–2343)
Marshall Islands	MHL	WPR	UMIC	1329 (553–2655)	5113 (2263–10189)
Macedonia, North	MKD	EUR	UMIC	664 (400–1007)	2554 (1566–3977)
Mali	MLI	AFR	LIC	346 (232–490)	1328 (906–1891)
Myanmar	MMR	SEAR	LMIC	428 (293–589)	1644 (1133–2343)
Montenegro	MNE	EUR	UMIC	833 (452–1376)	3204 (1787–5417)
Mongolia	MNG	WPR	LMIC	346 (232–490)	1328 (906–1891)
Mozambique	MOZ	AFR	LIC	833 (452–1376)	3204 (1787–5417)
Mauritania	MRT	AFR	LMIC	280 (178–415)	1078 (687–1598)
Malawi	MWI	AFR	LIC	664 (400–1007)	2554 (1566–3977)
Malaysia	MYS	WPR	UMIC	346 (232–490)	1328 (906–1891)
Namibia	NAM	AFR	UMIC	833 (452–1376)	3204 (1787–5417)
Niger	NER	AFR	LIC	532 (349–753)	2044 (1354–2999)
Nigeria	NGA	AFR	LMIC	280 (178–415)	1078 (687–1598)
Nicaragua	NIC	AMR	LMIC	833 (452–1376)	3204 (1787–5417)
Nepal	NPL	SEAR	LMIC	346 (232–490)	1328 (906–1891)
Pakistan	PAK	EMR	LMIC	280 (178–415)	1078 (687–1598)
Peru	PER	AMR	UMIC	428 (293–589)	1644 (1133–2343)

Country	Code	Region	Income level	Direct non-medical costs	
				RS	RR
Philippines	PHL	WPR	LMIC	346 (232–490)	1328 (906–1891)
Papua New Guinea	PNG	WPR	LMIC	229 (129–365)	878 (503–1397)
Korea, Democratic People's Republic of	PRK	SEAR	LIC	532 (349–753)	2044 (1354–2999)
Paraguay	PRY	AMR	UMIC	664 (400–1007)	2554 (1566–3977)
West Bank and Gaza	PSE	EMR	LMIC	428 (293–589)	1644 (1133–2343)
Russian Federation	RUS	EUR	UMIC	532 (349–753)	2044 (1354–2999)
Rwanda	RWA	AFR	LIC	532 (349–753)	2044 (1354–2999)
Sudan	SDN	EMR	LIC	428 (293–589)	1644 (1133–2343)
Senegal	SEN	AFR	LMIC	346 (232–490)	1328 (906–1891)
Solomon Islands	SLB	WPR	LMIC	428 (293–589)	1644 (1133–2343)
Sierra Leone	SLE	AFR	LIC	1050 (490–1903)	4038 (2002–7509)
El Salvador	SLV	AMR	LMIC	664 (400–1007)	2554 (1566–3977)
Somalia	SOM	EMR	LIC	532 (349–753)	2044 (1354–2999)
Serbia	SRB	EUR	UMIC	1050 (490–1903)	4038 (2002–7509)
South Sudan	SSD	AFR	LIC	532 (349–753)	2044 (1354–2999)
São Tomé and Principe	STP	AFR	LMIC	532 (349–753)	2044 (1354–2999)
Suriname	SUR	AMR	UMIC	1329 (553–2655)	5113 (2263–10189)
Swaziland	SWZ	AFR	LMIC	664 (400–1007)	2554 (1566–3977)

Country	Code	Region	Income level	Direct non-medical costs	
				RS	RR
Syrian Arab Republic	SYR	EMR	LIC	346 (232–490)	1328 (906–1891)
Chad	TCD	AFR	LIC	346 (232–490)	1328 (906–1891)
Togo	TGO	AFR	LIC	532 (349–753)	2044 (1354–2999)
Thailand	THA	SEAR	UMIC	346 (232–490)	1328 (906–1891)
Tajikistan	TJK	EUR	LMIC	664 (400–1007)	2554 (1566–3977)
Turkmenistan	TKM	EUR	UMIC	664 (400–1007)	2554 (1566–3977)
Timor-Leste	TLS	SEAR	LMIC	664 (400–1007)	2554 (1566–3977)
Tonga	TON	WPR	UMIC	428 (293–589)	1644 (1133–2343)
Tunisia	TUN	EMR	LMIC	664 (400–1007)	2554 (1566–3977)
Turkey	TUR	EUR	UMIC	346 (232–490)	1328 (906–1891)
Tuvalu	TUV	WPR	UMIC	1329 (553–2655)	5113 (2263–10189)
Tanzania, United Republic of	TZA	AFR	LMIC	346 (232–490)	1328 (906–1891)
Uganda	UGA	AFR	LIC	346 (232–490)	1328 (906–1891)
Ukraine	UKR	EUR	LMIC	664 (400–1007)	2554 (1566–3977)
Uzbekistan	UZB	EUR	LMIC	532 (349–753)	2044 (1354–2999)
St. Vincent and the Grenadines	VCT	AMR	UMIC	428 (293–589)	1644 (1133–2343)
Venezuela	VEN	AMR	UMIC	428 (293–589)	1644 (1133–2343)
Vietnam	VNM	WPR	LMIC	428 (293–589)	1644 (1133–2343)
Vanuatu	VUT	WPR	LMIC	280 (178–415)	1078 (687–1598)

Country	Code	Region	Income level	Direct non-medical costs	
				RS	RR
Samoa	WSM	WPR	LMIC	532 (349–753)	2044 (1354–2999)
Kosovo	XKX	EUR	UMIC	664 (400–1007)	2554 (1566–3977)
Yemen	YEM	EMR	LIC	346 (232–490)	1328 (906–1891)
South Africa	ZAF	AFR	UMIC	1050 (490–1903)	4038 (2002–7509)
Zambia	ZMB	AFR	LIC	428 (293–589)	1644 (1133–2343)
Zimbabwe	ZWE	AFR	LMIC	833 (452–1376)	3204 (1787–5417)

* All countries include 135 low- and middle-income countries analyzed. Values in parentheses represent equal-tailed 95% credible intervals.

Table S10. Predicted indirect unit cost for notified tuberculosis (TB) cases in 2021, stratified by rifampicin-sensitive (RS) and rifampicin-resistant (RR) by country.

Country	Code	Region	Income level	Indirect costs	
				RS	RR
Afghanistan	AFG	EMR	LIC	1185 (323–3111)	3556 (959–9729)
Angola	AGO	AFR	LMIC	311 (195–474)	932 (572–1435)
Albania	ALB	EUR	UMIC	668 (383–1070)	2003 (1150–3389)
Argentina	ARG	AMR	UMIC	2319 (813–5300)	6955 (2361–15502)
Armenia	ARM	EUR	UMIC	2144 (817–4677)	6433 (2484–13450)
American Samoa	ASM	WPR	UMIC	2066 (613–5118)	6186 (1817–14890)
Azerbaijan	AZE	EUR	UMIC	460 (256–769)	1378 (749–2305)
Burundi	BDI	AFR	LIC	699 (199–1710)	2099 (602–5420)
Benin	BEN	AFR	LMIC	216 (120–369)	647 (358–1081)
Burkina Faso	BFA	AFR	LIC	394 (239–644)	1182 (705–1885)
Bangladesh	BGD	SEAR	LMIC	231 (124–423)	691 (357–1240)
Bulgaria	BGR	EUR	UMIC	1091 (490–2167)	3268 (1438–6408)
Bosnia and Herzegovina	BIH	EUR	UMIC	2347 (868–5258)	7048 (2630–15333)
Belarus	BLR	EUR	UMIC	804 (447–1359)	2410 (1308–4168)
Belize	BLZ	AMR	UMIC	586 (278–1106)	1751 (795–3380)
Bolivia	BOL	AMR	LMIC	1026 (558–1701)	3077 (1688–5010)
Brazil	BRA	AMR	UMIC	1771 (644–3972)	5305 (1847–11275)
Bhutan	BTN	SEAR	LMIC	351 (201–605)	1050 (590–1743)

Country	Code	Region	Income level	Indirect costs	
				RS	RR
Botswana	BWA	AFR	UMIC	1023 (546–1748)	3070 (1618–5359)
Central African Republic	CAF	AFR	LIC	888 (308–2031)	2664 (947–6085)
China	CHN	WPR	UMIC	628 (256–1330)	1878 (770–3871)
Côte d'Ivoire	CIV	AFR	LMIC	332 (201–521)	995 (607–1544)
Cameroon	CMR	AFR	LMIC	421 (267–637)	1263 (791–1875)
Congo, Democratic Republic of the	COD	AFR	LIC	242 (125–443)	723 (367–1290)
Congo	COG	AFR	LMIC	266 (150–444)	795 (444–1366)
Colombia	COL	AMR	UMIC	1223 (615–2315)	3667 (1732–6561)
Comoros	COM	AFR	LMIC	755 (352–1446)	2264 (997–4121)
Cabo Verde	CPV	AFR	LMIC	390 (203–703)	1165 (601–2040)
Costa Rica	CRI	AMR	UMIC	2110 (741–4789)	6339 (2185–14849)
Cuba	CUB	AMR	UMIC	1812 (609–4170)	5427 (1764–11917)
Djibouti	DJI	EMR	LMIC	208 (92–441)	621 (256–1245)
Dominica	DMA	AMR	UMIC	1117 (459–2141)	3354 (1396–6702)
Dominican Republic	DOM	AMR	UMIC	902 (488–1575)	2706 (1407–4857)
Algeria	DZA	AFR	LMIC	633 (397–983)	1896 (1145–2923)
Ecuador	ECU	AMR	UMIC	1252 (634–2303)	3755 (1789–6672)
Egypt	EGY	EMR	LMIC	535 (340–806)	1602 (1044–2421)

Country	Code	Region	Income level	Indirect costs	
				RS	RR
Eritrea	ERI	AFR	LIC	307 (171–525)	921 (494–1546)
Ethiopia	ETH	AFR	LIC	209 (113–370)	624 (327–1079)
Fiji	FJI	WPR	UMIC	380 (198–686)	1137 (586–1997)
Micronesia	FSM	WPR	LMIC	1383 (541–2981)	4142 (1492–8691)
Gabon	GAB	AFR	UMIC	561 (258–1045)	1682 (783–3206)
Georgia	GEO	EUR	UMIC	958 (539–1626)	2873 (1566–4770)
Ghana	GHA	AFR	LMIC	493 (252–908)	1478 (733–2514)
Guinea	GIN	AFR	LIC	298 (184–479)	893 (544–1396)
Gambia	GMB	AFR	LIC	279 (161–473)	837 (472–1366)
Guinea-Bissau	GNB	AFR	LIC	1209 (410–2879)	3631 (1229–7953)
Equatorial Guinea	GNQ	AFR	UMIC	613 (277–1155)	1839 (844–3586)
Grenada	GRD	AMR	UMIC	1073 (472–2041)	3219 (1420–6328)
Guatemala	GTM	AMR	UMIC	795 (477–1243)	2382 (1427–3809)
Guyana	GUY	AMR	UMIC	722 (361–1287)	2164 (1080–4084)
Honduras	HND	AMR	LMIC	800 (474–1270)	2399 (1401–3730)
Haiti	HTI	AMR	LMIC	463 (307–672)	1387 (904–2029)
Indonesia	IDN	SEAR	LMIC	430 (241–687)	1287 (729–2152)
India	IND	SEAR	LMIC	287 (172–468)	860 (507–1383)
Iran	IRN	EMR	LMIC	951 (544–1550)	2852 (1586–4578)

Country	Code	Region	Income level	Indirect costs	
				RS	RR
Iraq	IRQ	EMR	UMIC	565 (330–897)	1694 (973–2720)
Jamaica	JAM	AMR	UMIC	557 (280–1022)	1666 (796–3125)
Jordan	JOR	EMR	UMIC	1328 (653–2430)	3986 (1937–7091)
Kazakhstan	KAZ	EUR	UMIC	377 (143–823)	1125 (403–2370)
Kenya	KEN	AFR	LMIC	539 (354–795)	1616 (1025–2351)
Kyrgyz Republic	KGZ	EUR	LMIC	321 (205–504)	962 (613–1482)
Cambodia	KHM	WPR	LMIC	696 (389–1111)	2085 (1172–3402)
Kiribati	KIR	WPR	LMIC	1250 (493–2668)	3746 (1399–7933)
Lao People's Democratic Republic	LAO	WPR	LMIC	255 (140–463)	762 (386–1378)
Lebanon	LBN	EMR	UMIC	1201 (544–2390)	3598 (1505–6752)
Liberia	LBR	AFR	LIC	1019 (377–2301)	3059 (1111–6474)
Libya	LBY	EMR	UMIC	1038 (553–1785)	3114 (1643–5480)
St. Lucia	LCA	AMR	UMIC	414 (153–904)	1237 (454–2668)
Sri Lanka	LKA	SEAR	LMIC	500 (299–757)	1498 (932–2333)
Lesotho	LSO	AFR	LMIC	2346 (601–6478)	7048 (1805–17370)
Morocco	MAR	EMR	LMIC	415 (218–742)	1241 (633–2145)
Moldova, Republic of	MDA	EUR	UMIC	768 (465–1220)	2301 (1359–3744)
Madagascar	MDG	AFR	LIC	292 (151–531)	876 (450–1546)

Country	Code	Region	Income level	Indirect costs	
				RS	RR
Maldives	MDV	SEAR	UMIC	1673 (717–3361)	5019 (2135–9938)
Mexico	MEX	AMR	UMIC	745 (378–1309)	2232 (1106–4222)
Marshall Islands	MHL	WPR	UMIC	2538 (917–5790)	7618 (2660–16788)
Macedonia, North	MKD	EUR	UMIC	1066 (558–1885)	3197 (1675–5578)
Mali	MLI	AFR	LIC	279 (162–463)	835 (490–1344)
Myanmar	MMR	SEAR	LMIC	403 (260–625)	1207 (760–1816)
Montenegro	MNE	EUR	UMIC	2016 (799–4309)	6054 (2360–12885)
Mongolia	MNG	WPR	LMIC	764 (354–1400)	2291 (1037–4157)
Mozambique	MOZ	AFR	LIC	534 (200–1177)	1598 (596–3501)
Mauritania	MRT	AFR	LMIC	305 (186–478)	914 (561–1425)
Malawi	MWI	AFR	LIC	636 (290–1210)	1908 (861–3582)
Malaysia	MYS	WPR	UMIC	463 (181–1020)	1383 (512–2892)
Namibia	NAM	AFR	UMIC	1302 (653–2322)	3906 (1938–6928)
Niger	NER	AFR	LIC	486 (243–879)	1458 (717–2627)
Nigeria	NGA	AFR	LMIC	469 (245–856)	1407 (710–2371)
Nicaragua	NIC	AMR	LMIC	891 (465–1538)	2672 (1359–4539)
Nepal	NPL	SEAR	LMIC	446 (252–735)	1336 (731–2139)
Pakistan	PAK	EMR	LMIC	325 (198–505)	974 (586–1500)
Peru	PER	AMR	UMIC	616 (341–1040)	1844 (1027–3161)

Country	Code	Region	Income level	Indirect costs	
				RS	RR
Philippines	PHL	WPR	LMIC	545 (328–852)	1634 (970–2543)
Papua New Guinea	PNG	WPR	LMIC	212 (104–422)	634 (287–1199)
Korea, Democratic People's Republic of	PRK	SEAR	LIC	432 (227–768)	1294 (666–2284)
Paraguay	PRY	AMR	UMIC	876 (470–1544)	2626 (1353–4558)
West Bank and Gaza	PSE	EMR	LMIC	401 (205–722)	1198 (600–2120)
Russian Federation	RUS	EUR	UMIC	710 (283–1540)	2123 (812–4540)
Rwanda	RWA	AFR	LIC	404 (213–717)	1210 (627–2125)
Sudan	SDN	EMR	LIC	364 (207–618)	1090 (618–1779)
Senegal	SEN	AFR	LMIC	326 (208–496)	975 (617–1463)
Solomon Islands	SLB	WPR	LMIC	412 (254–632)	1233 (751–1852)
Sierra Leone	SLE	AFR	LIC	873 (297–1995)	2618 (877–6303)
El Salvador	SLV	AMR	LMIC	977 (555–1606)	2931 (1619–4743)
Somalia	SOM	EMR	LIC	580 (232–1237)	1739 (689–3488)
Serbia	SRB	EUR	UMIC	2692 (914–6427)	8086 (2657–18716)
South Sudan	SSD	AFR	LIC	501 (292–811)	1503 (877–2404)
São Tomé and Principe	STP	AFR	LMIC	909 (456–1616)	2727 (1372–4681)
Suriname	SUR	AMR	UMIC	2035 (775–4379)	6104 (2388–12508)
Swaziland	SWZ	AFR	LMIC	930 (535–1517)	2789 (1567–4464)

Country	Code	Region	Income level	Indirect costs	
				RS	RR
Syrian Arab Republic	SYR	EMR	LIC	208 (98–417)	622 (281–1209)
Chad	TCD	AFR	LIC	316 (178–532)	948 (523–1581)
Togo	TGO	AFR	LIC	386 (202–682)	1155 (591–2036)
Thailand	THA	SEAR	UMIC	381 (154–800)	1138 (452–2331)
Tajikistan	TJK	EUR	LMIC	726 (350–1331)	2179 (1048–3878)
Turkmenistan	TKM	EUR	UMIC	958 (492–1715)	2869 (1442–5210)
Timor-Leste	TLS	SEAR	LMIC	993 (541–1682)	2980 (1617–4894)
Tonga	TON	WPR	UMIC	455 (243–799)	1362 (713–2336)
Tunisia	TUN	EMR	LMIC	866 (510–1406)	2596 (1482–4114)
Turkey	TUR	EUR	UMIC	493 (215–993)	1474 (641–2840)
Tuvalu	TUV	WPR	UMIC	1553 (580–3427)	4649 (1588–9732)
Tanzania, United Republic of	TZA	AFR	LMIC	302 (187–487)	903 (558–1410)
Uganda	UGA	AFR	LIC	272 (156–456)	813 (472–1338)
Ukraine	UKR	EUR	LMIC	1024 (570–1697)	3071 (1673–5134)
Uzbekistan	UZB	EUR	LMIC	640 (394–967)	1920 (1173–2847)
St. Vincent and the Grenadines	VCT	AMR	UMIC	508 (210–1051)	1519 (624–3125)
Venezuela	VEN	AMR	UMIC	722 (280–1556)	2160 (865–4507)
Vietnam	VNM	WPR	LMIC	561 (361–829)	1681 (1086–2535)
Vanuatu	VUT	WPR	LMIC	277 (150–497)	828 (424–1455)

Country	Code	Region	Income level	Indirect costs	
				RS	RR
Samoa	WSM	WPR	LMIC	515 (273–902)	1540 (768–2718)
Kosovo	XKX	EUR	UMIC	970 (539–1655)	2909 (1572–4886)
Yemen	YEM	EMR	LIC	286 (162–486)	857 (471–1415)
South Africa	ZAF	AFR	UMIC	1802 (743–3652)	5407 (2237–10738)
Zambia	ZMB	AFR	LIC	364 (219–602)	1091 (671–1693)
Zimbabwe	ZWE	AFR	LMIC	1038 (519–1917)	3113 (1532–5442)

* All countries include 135 low- and middle-income countries analyzed. Values in parentheses represent equal-tailed 95% credible intervals.

Table S11. Predicted direct medical, direct non-medical, and indirect unit cost overall for notified tuberculosis (TB) cases in 2021.

Country	Code	Region	Income level	Direct medical costs	Direct non-medical costs	Indirect costs
Afghanistan	AFG	EMR	LIC	732 (322–1436)	1201 (598–2205)	2286 (1329–3608)
Angola	AGO	AFR	LMIC	122 (84–173)	267 (212–331)	373 (289–482)
Albania	ALB	EUR	UMIC	232 (154–338)	1062 (738–1449)	492 (394–615)
Argentina	ARG	AMR	UMIC	1822 (717–3837)	3269 (1860–5330)	3215 (1685–5632)
Armenia	ARM	EUR	UMIC	2179 (877–4545)	3059 (1829–4816)	3611 (1950–6259)
American Samoa	ASM	WPR	UMIC	324 (141–643)	2874 (1599–4882)	1153 (647–1895)
Azerbaijan	AZE	EUR	UMIC	474 (200–961)	723 (510–983)	643 (387–1037)
Burundi	BDI	AFR	LIC	596 (351–954)	886 (499–1513)	1397 (950–1938)
Benin	BEN	AFR	LMIC	182 (91–322)	356 (239–504)	279 (199–386)
Burkina Faso	BFA	AFR	LIC	241 (166–340)	640 (470–846)	501 (414–606)
Bangladesh	BGD	SEAR	LMIC	62 (39–92)	264 (196–345)	183 (131–245)
Bulgaria	BGR	EUR	UMIC	611 (342–1024)	1641 (1066–2445)	1119 (753–1637)
Bosnia and Herzegovina	BIH	EUR	UMIC	658 (232–1439)	3336 (1969–5312)	1613 (791–2899)
Belarus	BLR	EUR	UMIC	765 (363–1472)	1256 (888–1733)	1112 (689–1742)
Belize	BLZ	AMR	UMIC	149 (99–216)	914 (653–1296)	422 (311–558)
Bolivia	BOL	AMR	LMIC	431 (253–691)	1387 (1014–1824)	1019 (716–1394)
Brazil	BRA	AMR	UMIC	554 (259–1049)	2540 (1511–4085)	1588 (912–2534)
Bhutan	BTN	SEAR	LMIC	302 (124–625)	441 (351–557)	559 (309–967)

Country	Code	Region	Income level	Direct medical costs	Direct non-medical costs	Indirect costs
Botswana	BWA	AFR	UMIC	304 (196–454)	1040 (743–1388)	855 (634–1134)
Central African Republic	CAF	AFR	LIC	209 (66–502)	464 (212–895)	1260 (776–1923)
China	CHN	WPR	UMIC	457 (179–941)	937 (604–1392)	715 (402–1233)
Côte d'Ivoire	CIV	AFR	LMIC	373 (169–719)	452 (344–565)	541 (334–857)
Cameroon	CMR	AFR	LMIC	170 (117–240)	520 (414–630)	426 (329–551)
Congo, Democratic Republic of the	COD	AFR	LIC	91 (63–129)	221 (149–309)	339 (268–430)
Congo	COG	AFR	LMIC	80 (52–117)	214 (161–285)	264 (197–348)
Colombia	COL	AMR	UMIC	392 (224–636)	1848 (1273–2600)	1006 (680–1414)
Comoros	COM	AFR	LMIC	363 (138–793)	1171 (694–1801)	664 (355–1133)
Cabo Verde	CPV	AFR	LMIC	121 (80–176)	627 (465–856)	324 (242–425)
Costa Rica	CRI	AMR	UMIC	589 (198–1412)	3016 (1600–5145)	1168 (558–2109)
Cuba	CUB	AMR	UMIC	431 (200–823)	2578 (1503–4273)	1365 (786–2204)
Djibouti	DJI	EMR	LMIC	64 (37–107)	231 (159–328)	184 (123–268)
Dominica	DMA	AMR	UMIC	362 (138–790)	1692 (933–2698)	664 (355–1133)
Dominican Republic	DOM	AMR	UMIC	729 (387–1305)	1399 (950–1977)	1091 (748–1585)
Algeria	DZA	AFR	LMIC	264 (189–359)	978 (768–1245)	613 (491–757)
Ecuador	ECU	AMR	UMIC	706 (413–1164)	1879 (1303–2632)	1423 (962–2025)
Egypt	EGY	EMR	LMIC	192 (132–274)	864 (650–1110)	439 (351–544)

Country	Code	Region	Income level	Direct medical costs	Direct non-medical costs	Indirect costs
Eritrea	ERI	AFR	LIC	240 (162–340)	466 (331–631)	456 (378–554)
Ethiopia	ETH	AFR	LIC	92 (61–135)	288 (214–372)	236 (179–309)
Fiji	FJI	WPR	UMIC	90 (58–136)	588 (428–788)	247 (185–324)
Micronesia	FSM	WPR	LMIC	305 (132–621)	1893 (1151–3067)	1153 (647–1895)
Gabon	GAB	AFR	UMIC	108 (61–181)	314 (196–475)	438 (340–574)
Georgia	GEO	EUR	UMIC	955 (464–1779)	1405 (1031–1875)	1510 (921–2429)
Ghana	GHA	AFR	LMIC	255 (132–450)	639 (406–921)	461 (308–676)
Guinea	GIN	AFR	LIC	254 (134–439)	369 (299–448)	522 (342–783)
Gambia	GMB	AFR	LIC	281 (150–477)	361 (280–457)	540 (356–799)
Guinea-Bissau	GNB	AFR	LIC	776 (364–1487)	888 (489–1456)	2279 (1409–3440)
Equatorial Guinea	GNQ	AFR	UMIC	434 (229–750)	577 (385–812)	735 (468–1095)
Grenada	GRD	AMR	UMIC	319 (132–654)	1639 (930–2581)	612 (339–1007)
Guatemala	GTM	AMR	UMIC	306 (201–452)	1252 (911–1674)	666 (515–852)
Guyana	GUY	AMR	UMIC	188 (130–264)	1047 (703–1492)	454 (361–566)
Honduras	HND	AMR	LMIC	741 (420–1259)	1254 (947–1606)	1263 (855–1870)
Haiti	HTI	AMR	LMIC	223 (173–280)	576 (488–682)	558 (463–667)
Indonesia	IDN	SEAR	LMIC	112 (75–153)	400 (307–511)	340 (261–442)
India	IND	SEAR	LMIC	138 (92–200)	349 (282–428)	325 (245–431)
Iran	IRN	EMR	LMIC	297 (162–500)	1476 (1083–1957)	753 (503–1089)

Country	Code	Region	Income level	Direct medical costs	Direct non-medical costs	Indirect costs
Iraq	IRQ	EMR	UMIC	698 (309–1357)	912 (598–1285)	809 (526–1204)
Jamaica	JAM	AMR	UMIC	121 (78–186)	874 (635–1225)	375 (269–508)
Jordan	JOR	EMR	UMIC	570 (297–969)	2000 (1368–2787)	1273 (844–1811)
Kazakhstan	KAZ	EUR	UMIC	288 (103–662)	561 (337–863)	415 (221–749)
Kenya	KEN	AFR	LMIC	185 (135–247)	547 (455–651)	569 (457–711)
Kyrgyz Republic	KGZ	EUR	LMIC	426 (189–837)	466 (371–577)	643 (387–1037)
Cambodia	KHM	WPR	LMIC	207 (125–323)	670 (475–910)	797 (589–1050)
Kiribati	KIR	WPR	LMIC	454 (139–1120)	836 (378–1611)	2172 (1202–3657)
Lao People's Democratic Republic	LAO	WPR	LMIC	73 (49–109)	335 (253–439)	210 (156–275)
Lebanon	LBN	EMR	UMIC	318 (162–572)	1795 (1190–2621)	1005 (626–1527)
Liberia	LBR	AFR	LIC	332 (140–681)	842 (480–1370)	1300 (754–2070)
Libya	LBY	EMR	UMIC	462 (268–752)	1552 (1024–2195)	876 (637–1184)
St. Lucia	LCA	AMR	UMIC	76 (48–119)	645 (405–993)	216 (158–291)
Sri Lanka	LKA	SEAR	LMIC	152 (85–252)	779 (547–1039)	346 (244–489)
Lesotho	LSO	AFR	LMIC	729 (172–1937)	1119 (446–2305)	3497 (1788–5979)
Morocco	MAR	EMR	LMIC	219 (117–359)	593 (450–790)	488 (324–719)
Moldova, Republic of	MDA	EUR	UMIC	766 (382–1436)	1135 (849–1507)	1161 (737–1823)
Madagascar	MDG	AFR	LIC	102 (68–148)	313 (226–417)	340 (254–453)

Country	Code	Region	Income level	Direct medical costs	Direct non-medical costs	Indirect costs
Maldives	MDV	SEAR	UMIC	465 (229–811)	2460 (1552–3734)	1130 (710–1703)
Mexico	MEX	AMR	UMIC	254 (171–359)	1164 (752–1702)	518 (427–630)
Marshall Islands	MHL	WPR	UMIC	291 (71–817)	1427 (671–2750)	1860 (910–3335)
Macedonia, North	MKD	EUR	UMIC	311 (179–500)	1636 (1156–2288)	772 (532–1080)
Mali	MLI	AFR	LIC	224 (139–346)	454 (337–598)	414 (315–535)
Myanmar	MMR	SEAR	LMIC	177 (126–249)	373 (285–484)	575 (462–708)
Montenegro	MNE	EUR	UMIC	538 (208–1156)	2916 (1734–4606)	1253 (647–2176)
Mongolia	MNG	WPR	LMIC	391 (216–663)	507 (337–719)	977 (648–1405)
Mozambique	MOZ	AFR	LIC	124 (55–260)	415 (204–757)	727 (488–1028)
Mauritania	MRT	AFR	LMIC	96 (56–149)	463 (343–597)	234 (169–321)
Malawi	MWI	AFR	LIC	328 (207–496)	797 (549–1157)	900 (651–1201)
Malaysia	MYS	WPR	UMIC	91 (62–133)	649 (416–982)	255 (193–331)
Namibia	NAM	AFR	UMIC	477 (196–1037)	819 (469–1359)	1847 (1196–2790)
Niger	NER	AFR	LIC	464 (311–692)	706 (495–997)	887 (680–1145)
Nigeria	NGA	AFR	LMIC	306 (177–482)	515 (353–714)	563 (404–788)
Nicaragua	NIC	AMR	LMIC	318 (175–526)	1372 (985–1869)	887 (588–1270)
Nepal	NPL	SEAR	LMIC	217 (147–310)	475 (362–610)	536 (411–706)
Pakistan	PAK	EMR	LMIC	159 (112–212)	337 (272–417)	394 (310–505)
Peru	PER	AMR	UMIC	453 (242–803)	837 (623–1097)	781 (519–1160)

Country	Code	Region	Income level	Direct medical costs	Direct non-medical costs	Indirect costs
Philippines	PHL	WPR	LMIC	137 (74–233)	305 (200–441)	582 (463–733)
Papua New Guinea	PNG	WPR	LMIC	27 (14–48)	150 (99–225)	145 (101–202)
Korea, Democratic People's Republic of	PRK	SEAR	LIC	68 (27–143)	254 (140–443)	475 (344–646)
Paraguay	PRY	AMR	UMIC	389 (252–571)	1347 (972–1829)	866 (636–1151)
West Bank and Gaza	PSE	EMR	LMIC	96 (62–145)	643 (471–881)	284 (209–382)
Russian Federation	RUS	EUR	UMIC	499 (191–1071)	1074 (695–1647)	843 (449–1508)
Rwanda	RWA	AFR	LIC	170 (116–243)	630 (460–848)	470 (361–606)
Sudan	SDN	EMR	LIC	244 (173–333)	568 (422–748)	514 (426–618)
Senegal	SEN	AFR	LMIC	108 (75–150)	460 (376–558)	296 (232–375)
Solomon Islands	SLB	WPR	LMIC	118 (78–176)	642 (514–796)	334 (254–439)
Sierra Leone	SLE	AFR	LIC	347 (161–672)	750 (385–1318)	1444 (919–2111)
El Salvador	SLV	AMR	LMIC	336 (198–528)	1481 (1083–1975)	819 (571–1134)
Somalia	SOM	EMR	LIC	754 (422–1261)	557 (351–849)	1488 (961–2229)
Serbia	SRB	EUR	UMIC	729 (248–1666)	3767 (2132–6197)	1722 (816–3198)
South Sudan	SSD	AFR	LIC	202 (144–277)	537 (405–699)	653 (521–814)
São Tomé and Principe	STP	AFR	LMIC	487 (273–817)	1197 (834–1644)	984 (667–1410)
Suriname	SUR	AMR	UMIC	2036 (813–4336)	2916 (1752–4601)	3456 (1836–5999)
Swaziland	SWZ	AFR	LMIC	383 (221–635)	803 (570–1113)	1210 (862–1662)

Country	Code	Region	Income level	Direct medical costs	Direct non-medical costs	Indirect costs
Syrian Arab Republic	SYR	EMR	LIC	102 (65–156)	340 (227–482)	255 (190–340)
Chad	TCD	AFR	LIC	465 (226–858)	417 (313–543)	720 (457–1113)
Togo	TGO	AFR	LIC	162 (111–232)	619 (446–833)	445 (336–576)
Thailand	THA	SEAR	UMIC	110 (67–169)	478 (327–701)	303 (208–430)
Tajikistan	TJK	EUR	LMIC	1108 (556–2019)	1030 (722–1438)	1696 (1067–2618)
Turkmenistan	TKM	EUR	UMIC	216 (127–352)	1464 (1026–2028)	619 (425–876)
Timor-Leste	TLS	SEAR	LMIC	163 (62–351)	575 (336–919)	923 (601–1360)
Tonga	TON	WPR	UMIC	110 (72–165)	730 (544–981)	310 (233–410)
Tunisia	TUN	EMR	LMIC	294 (178–458)	1351 (1006–1757)	742 (527–1009)
Turkey	TUR	EUR	UMIC	163 (107–239)	778 (501–1156)	341 (272–428)
Tuvalu	TUV	WPR	UMIC	243 (91–557)	1329 (693–2355)	1298 (744–2084)
Tanzania, United Republic of	TZA	AFR	LMIC	94 (69–126)	340 (274–415)	312 (247–390)
Uganda	UGA	AFR	LIC	112 (86–145)	323 (251–406)	332 (269–413)
Ukraine	UKR	EUR	LMIC	1060 (527–1955)	1486 (1085–1989)	1623 (1008–2541)
Uzbekistan	UZB	EUR	LMIC	853 (424–1594)	975 (751–1225)	1230 (793–1860)
St. Vincent and the Grenadines	VCT	AMR	UMIC	96 (62–145)	790 (525–1175)	284 (209–382)
Venezuela	VEN	AMR	UMIC	152 (105–215)	1096 (674–1683)	378 (302–471)
Vietnam	VNM	WPR	LMIC	498 (252–890)	679 (561–810)	894 (570–1365)
Vanuatu	VUT	WPR	LMIC	78 (47–124)	457 (326–623)	193 (139–262)

Country	Code	Region	Income level	Direct medical costs	Direct non-medical costs	Indirect costs
Samoa	WSM	WPR	LMIC	121 (78–186)	815 (607–1116)	375 (269–508)
Kosovo	XKX	EUR	UMIC	314 (189–492)	1501 (1086–2033)	773 (547–1062)
Yemen	YEM	EMR	LIC	189 (126–277)	467 (333–638)	378 (313–453)
South Africa	ZAF	AFR	UMIC	317 (106–793)	940 (444–1801)	1799 (1130–2646)
Zambia	ZMB	AFR	LIC	179 (112–284)	331 (242–444)	581 (421–791)
Zimbabwe	ZWE	AFR	LMIC	377 (204–649)	1147 (785–1604)	1194 (797–1693)

* All countries include 135 low- and middle-income countries analyzed. Values in parentheses represent equal-tailed 95% credible intervals.

Table S12. Predicted proportion of households experiencing catastrophic costs due to tuberculosis, stratified by income quintile*.

Country	Code	Region	Income level	Poorest	Poorer	Middle	Richer	Richest	Overall
Afghanistan	AFG	EMR	LIC	97·34% (95·43–98·72%)	97·40% (94·35–99·23%)	95·71% (90·86–98·64%)	94·54% (88·34–98·39%)	86·95% (77·98–93·66%)	94·39% (89·39–97·73%)
Angola	AGO	AFR	LMIC	73·41% (68·78–78·07%)	56·84% (48·72–65·04%)	47·69% (39·34–56·65%)	42·76% (33·05–53·80%)	40·10% (32·41–49·19%)	52·16% (44·46–60·55%)
Albania	ALB	EUR	UMIC	78·82% (73·51–83·90%)	63·84% (53·92–73·00%)	54·52% (43·79–64·92%)	46·70% (35·27–57·56%)	42·17% (33·44–52·14%)	57·21% (47·98–66·31%)
Argentina	ARG	AMR	UMIC	86·28% (82·04–89·99%)	77·97% (70·50–84·69%)	70·10% (61·13–78·54%)	64·53% (52·99–75·69%)	56·95% (48·77–66·26%)	71·16% (63·08–79·03%)
Armenia	ARM	EUR	UMIC	91·04% (88·24–93·39%)	86·82% (82·44–90·59%)	80·77% (74·22–86·50%)	76·49% (67·95–84·77%)	67·30% (59·92–75·30%)	80·48% (74·55–86·11%)
American Samoa	ASM	WPR	UMIC	86·95% (81·54–91·65%)	78·45% (66·07–88·37%)	71·71% (57·22–83·96%)	64·67% (47·55–80·01%)	56·04% (42·23–70·47%)	71·56% (58·92–82·89%)
Azerbaijan	AZE	EUR	UMIC	66·37% (58·07–74·52%)	44·42% (33·36–57·92%)	34·49% (23·96–46·79%)	28·76% (18·75–41·49%)	29·30% (20·26–39·40%)	40·67% (30·88–52·02%)
Burundi	BDI	AFR	LIC	96·49% (93·94–98·38%)	96·14% (91·95–98·75%)	93·69% (87·16–97·87%)	92·02% (83·93–97·19%)	83·54% (73·78–91·38%)	92·38% (86·15–96·71%)
Benin	BEN	AFR	LMIC	70·90% (65·61–76·35%)	51·12% (43·02–60·21%)	40·97% (32·56–50·73%)	34·58% (26·05–45·81%)	33·69% (25·60–43·19%)	46·25% (38·57–55·26%)
Burkina Faso	BFA	AFR	LIC	86·38% (82·80–89·89%)	77·96% (71·76–83·46%)	70·22% (61·80–78·13%)	64·24% (54·48–73·48%)	56·42% (47·74–65·14%)	71·04% (63·72–78·02%)
Bangladesh	BGD	SEAR	LMIC	67·64% (64·85–70·67%)	46·87% (40·65–53·81%)	37·40% (30·41–44·60%)	31·22% (23·37–40·00%)	30·63% (23·89–38·67%)	42·75% (36·63–49·55%)
Bulgaria	BGR	EUR	UMIC	74·76% (68·84–80·53%)	57·29% (47·31–67·39%)	47·43% (37·06–58·18%)	40·57% (29·69–52·58%)	37·97% (29·93–47·32%)	51·61% (42·56–61·20%)
Bosnia and Herzegovina	BIH	EUR	UMIC	94·15% (89·94–97·31%)	91·65% (84·57–96·82%)	87·86% (77·78–94·89%)	83·49% (70·63–93·21%)	73·43% (60·18–84·67%)	86·12% (76·62–93·38%)
Belarus	BLR	EUR	UMIC	73·98% (67·02–80·10%)	56·13% (45·66–66·98%)	46·01% (35·53–57·09%)	39·65% (28·74–52·00%)	37·70% (29·20–47·03%)	50·69% (41·23–60·64%)
Belize	BLZ	AMR	UMIC	74·90% (69·87–79·78%)	57·52% (46·70–68·42%)	48·09% (36·93–59·92%)	40·49% (28·71–53·26%)	37·26% (27·73–48·56%)	51·65% (41·99–61·99%)
Bolivia	BOL	AMR	LMIC	89·44% (85·97–92·47%)	83·57% (78·36–88·72%)	77·19% (69·95–83·93%)	71·75% (61·65–80·58%)	62·53% (53·47–71·57%)	76·90% (69·88–83·46%)
Brazil	BRA	AMR	UMIC	88·29% (85·67–91·03%)	81·27% (74·54–88·03%)	74·68% (66·05–82·93%)	68·72% (57·37–80·42%)	59·73% (50·19–70·51%)	74·54% (66·76–82·59%)

Country	Code	Region	Income level	Poorest	Poorer	Middle	Richer	Richest	Overall
Bhutan	BTN	SEAR	LMIC	64·97% (56·43–74·10%)	43·26% (30·88–58·40%)	33·89% (22·34–48·53%)	29·08% (17·28–44·25%)	29·73% (20·01–41·61%)	40·19% (29·39–53·38%)
Botswana	BWA	AFR	UMIC	83·90% (77·89–88·89%)	73·92% (64·83–81·92%)	65·95% (55·75–75·76%)	59·93% (47·60–71·42%)	52·85% (43·06–62·51%)	67·31% (57·82–76·10%)
Central African Republic	CAF	AFR	LIC	96·66% (94·39–98·28%)	96·78% (93·48–98·90%)	95·06% (89·61–98·38%)	94·03% (87·11–98·24%)	86·14% (76·66–93·44%)	93·73% (88·25–97·45%)
China	CHN	WPR	UMIC	60·33% (51·34–69·31%)	36·26% (24·91–49·45%)	27·10% (17·13–39·87%)	21·87% (12·22–34·60%)	23·53% (15·24–33·53%)	33·82% (24·17–45·35%)
Côte d'Ivoire	CIV	AFR	LMIC	69·15% (60·23–78·21%)	49·07% (36·22–63·91%)	39·12% (26·45–53·75%)	33·71% (21·32–48·75%)	33·44% (23·27–45·55%)	44·90% (33·50–58·03%)
Cameroon	CMR	AFR	LMIC	83·52% (79·65–86·82%)	73·05% (67·73–78·19%)	64·73% (57·93–71·92%)	58·53% (49·69–67·15%)	51·79% (44·32–59·77%)	66·32% (59·86–72·77%)
Congo, Democratic Republic of the	COD	AFR	LIC	80·90% (75·87–86·20%)	69·77% (58·91–79·34%)	61·75% (50·41–73·67%)	57·27% (44·87–69·79%)	51·13% (40·83–61·95%)	64·16% (54·18–74·19%)
Congo	COG	AFR	LMIC	69·39% (65·06–73·86%)	50·47% (43·25–58·03%)	41·34% (33·87–49·64%)	36·35% (27·49–47·34%)	35·10% (27·48–43·43%)	46·53% (39·43–54·46%)
Colombia	COL	AMR	UMIC	87·30% (83·80–90·54%)	79·28% (72·12–86·01%)	72·18% (63·58–80·79%)	65·62% (53·66–76·45%)	57·12% (47·56–67·16%)	72·30% (64·14–80·19%)
Comoros	COM	AFR	LMIC	92·54% (87·43–96·31%)	88·96% (80·87–94·94%)	83·98% (73·20–91·97%)	79·06% (65·04–89·14%)	69·23% (56·95–80·35%)	82·76% (72·70–90·54%)
Cabo Verde	CPV	AFR	LMIC	73·77% (69·17–78·40%)	55·75% (46·09–65·46%)	46·18% (36·72–56·44%)	38·84% (28·30–50·15%)	36·16% (27·49–46·77%)	50·14% (41·55–59·45%)
Costa Rica	CRI	AMR	UMIC	91·12% (83·20–96·44%)	86·02% (72·43–94·99%)	80·59% (63·43–92·47%)	74·60% (55·21–89·33%)	65·04% (48·78–79·84%)	79·47% (64·61–90·62%)
Cuba	CUB	AMR	UMIC	87·61% (84·04–90·97%)	79·87% (70·90–87·81%)	73·17% (62·41–83·04%)	66·76% (53·34–79·70%)	57·96% (46·89–70·10%)	73·07% (63·51–82·33%)
Djibouti	DJI	EMR	LMIC	57·69% (52·31–62·97%)	33·67% (25·53–43·78%)	25·16% (17·55–34·43%)	20·43% (12·65–30·65%)	22·23% (15·00–31·49%)	31·84% (24·61–40·67%)
Dominica	DMA	AMR	UMIC	87·71% (79·18–94·00%)	79·71% (64·96–90·52%)	72·70% (55·42–86·73%)	65·60% (46·79–81·54%)	57·22% (42·47–71·52%)	72·59% (57·76–84·86%)
Dominican Republic	DOM	AMR	UMIC	75·71% (69·23–81·79%)	58·87% (48·47–69·12%)	48·81% (38·48–59·47%)	42·12% (31·13–54·72%)	39·45% (31·10–48·33%)	52·99% (43·68–62·69%)
Algeria	DZA	AFR	LMIC	81·24% (78·54–83·82%)	68·41% (63·03–73·94%)	59·48% (52·67–66·76%)	52·44% (43·87–61·28%)	46·82% (39·54–55·02%)	61·68% (55·53–68·16%)
Ecuador	ECU	AMR	UMIC	85·38% (82·02–88·34%)	76·05% (70·31–81·35%)	67·97% (60·67–74·86%)	61·71% (52·23–70·91%)	54·37% (47·11–62·88%)	69·09% (62·47–75·67%)
Egypt	EGY	EMR	LMIC	80·44% (76·59–84·18%)	66·76% (59·86–73·36%)	57·71% (49·40–66·22%)	50·12% (40·75–59·46%)	44·84% (36·97–54·02%)	59·97% (52·71–67·45%)

Country	Code	Region	Income level	Poorest	Poorer	Middle	Richer	Richest	Overall
Eritrea	ERI	AFR	LIC	84·70% (80·28–88·92%)	75·15% (67·20–82·45%)	66·79% (57·15–76·18%)	61·07% (49·94–71·19%)	54·15% (45·76–63·45%)	68·37% (60·06–76·44%)
Ethiopia	ETH	AFR	LIC	74·82% (69·68–79·80%)	58·01% (48·67–66·68%)	48·52% (39·26–59·01%)	42·18% (32·28–53·28%)	39·24% (30·10–48·72%)	52·56% (44·00–61·50%)
Fiji	FJI	WPR	UMIC	72·42% (67·00–77·81%)	53·53% (43·18–64·06%)	43·94% (32·67–55·15%)	36·27% (25·45–48·00%)	33·94% (25·16–44·65%)	48·02% (38·69–57·94%)
Micronesia	FSM	WPR	LMIC	91·62% (88·91–94·35%)	87·39% (80·97–93·41%)	82·55% (74·33–90·29%)	77·59% (66·59–88·24%)	67·44% (56·45–78·04%)	81·32% (73·45–88·87%)
Gabon	GAB	AFR	UMIC	68·91% (59·39–77·49%)	50·63% (38·78–62·36%)	41·91% (31·00–53·56%)	37·39% (25·84–50·48%)	35·89% (27·01–45·02%)	46·95% (36·40–57·78%)
Georgia	GEO	EUR	UMIC	80·99% (75·58–85·52%)	68·44% (59·77–76·51%)	59·06% (49·69–67·96%)	53·12% (42·63–64·16%)	48·13% (40·42–56·43%)	61·95% (53·62–70·12%)
Ghana	GHA	AFR	LMIC	83·45% (76·38–89·19%)	72·66% (62·46–81·61%)	64·01% (52·87–74·48%)	57·42% (44·33–68·75%)	51·05% (40·82–61·04%)	65·72% (55·37–75·01%)
Guinea	GIN	AFR	LIC	75·40% (68·72–82·22%)	59·51% (47·07–71·48%)	49·98% (37·92–62·70%)	44·81% (32·62–58·86%)	41·88% (32·07–53·50%)	54·32% (43·68–65·75%)
Gambia	GMB	AFR	LIC	78·74% (71·97–85·55%)	65·18% (51·97–77·32%)	55·87% (42·48–69·05%)	50·79% (37·31–65·55%)	46·54% (35·64–58·92%)	59·42% (47·87–71·28%)
Guinea-Bissau	GNB	AFR	LIC	95·92% (93·35–97·74%)	95·68% (92·15–98·02%)	93·08% (87·45–96·97%)	91·63% (84·24–96·65%)	83·12% (75·10–90·32%)	91·89% (86·46–95·94%)
Equatorial Guinea	GNQ	AFR	UMIC	69·86% (57·55–80·82%)	50·75% (34·66–67·86%)	41·04% (26·12–57·03%)	35·82% (21·49–52·21%)	35·06% (23·88–47·21%)	46·51% (32·74–61·03%)
Grenada	GRD	AMR	UMIC	85·94% (77·29–92·74%)	76·44% (61·37–88·06%)	68·83% (51·17–83·69%)	61·28% (42·70–77·68%)	53·59% (39·00–68·04%)	69·22% (54·31–82·04%)
Guatemala	GTM	AMR	UMIC	83·49% (79·30–87·41%)	72·24% (64·71–79·15%)	63·78% (54·76–72·46%)	56·51% (45·46–66·45%)	49·91% (41·25–59·13%)	65·18% (57·09–72·92%)
Guyana	GUY	AMR	UMIC	75·84% (69·09–82·39%)	58·95% (46·78–70·44%)	49·47% (37·27–62·56%)	41·65% (28·83–55·03%)	38·17% (28·33–49·38%)	52·82% (42·06–63·96%)
Honduras	HND	AMR	LMIC	84·39% (81·10–87·35%)	74·48% (69·05–79·63%)	65·86% (58·85–72·40%)	60·05% (51·28–68·64%)	53·40% (46·92–60·83%)	67·64% (61·44–73·77%)
Haiti	HTI	AMR	LMIC	82·02% (79·23–84·60%)	70·52% (65·70–74·99%)	61·88% (55·02–68·57%)	55·97% (47·48–64·47%)	49·93% (42·87–57·66%)	64·06% (58·06–70·06%)
Indonesia	IDN	SEAR	LMIC	74·03% (68·63–79·06%)	57·10% (49·15–64·34%)	47·76% (39·45–56·43%)	41·59% (31·96–51·78%)	38·73% (31·17–47·01%)	51·84% (44·07–59·73%)
India	IND	SEAR	LMIC	70·02% (66·29–74·14%)	50·38% (43·54–57·97%)	40·72% (33·55–48·92%)	34·90% (26·63–45·00%)	33·88% (26·61–42·14%)	45·98% (39·32–53·63%)
Iran	IRN	EMR	LMIC	88·46% (84·66–91·85%)	81·35% (74·03–87·97%)	74·67% (65·94–82·81%)	68·22% (56·95–78·60%)	59·25% (49·15–69·21%)	74·39% (66·14–82·09%)

Country	Code	Region	Income level	Poorest	Poorer	Middle	Richer	Richest	Overall
Iraq	IRQ	EMR	UMIC	73·78% (64·70–81·90%)	55·75% (42·68–68·78%)	45·37% (32·75–57·69%)	38·94% (26·95–52·65%)	37·34% (27·81–47·52%)	50·24% (38·98–61·71%)
Jamaica	JAM	AMR	UMIC	77·46% (71·95–82·59%)	61·77% (50·43–72·69%)	52·68% (40·51–65·20%)	44·84% (32·19–58·59%)	40·48% (30·00–52·60%)	55·45% (45·01–66·33%)
Jordan	JOR	EMR	UMIC	90·84% (87·43–93·74%)	85·95% (80·47–91·14%)	80·17% (72·70–86·99%)	74·82% (64·35–83·82%)	65·18% (55·80–74·73%)	79·39% (72·15–86·08%)
Kazakhstan	KAZ	EUR	UMIC	51·14% (41·67–61·80%)	25·60% (16·10–39·26%)	17·89% (10·09–29·60%)	13·89% (6·84–25·18%)	16·67% (9·43–26·51%)	25·04% (16·83–36·47%)
Kenya	KEN	AFR	LMIC	84·45% (81·04–87·09%)	75·23% (70·54–79·94%)	67·46% (60·78–73·79%)	62·06% (53·19–70·17%)	54·64% (47·11–62·47%)	68·77% (62·53–74·69%)
Kyrgyz Republic	KGZ	EUR	LMIC	75·67% (67·70–83·47%)	59·62% (46·32–73·08%)	49·72% (35·83–64·23%)	44·31% (30·58–59·66%)	41·68% (31·07–54·24%)	54·20% (42·30–66·94%)
Cambodia	KHM	WPR	LMIC	88·96% (86·60–91·19%)	83·56% (78·91–88·23%)	77·61% (70·79–83·90%)	73·32% (64·27–81·99%)	63·95% (55·47–72·45%)	77·48% (71·21–83·55%)
Kiribati	KIR	WPR	LMIC	92·21% (89·52–94·58%)	90·11% (84·76–94·38%)	86·07% (78·63–92·09%)	83·98% (74·48–91·58%)	74·32% (64·56–83·33%)	85·34% (78·39–91·19%)
Lao People's Democratic Republic	LAO	WPR	LMIC	68·47% (64·73–72·56%)	47·91% (39·83–56·51%)	38·37% (30·33–47·36%)	31·90% (23·36–41·65%)	31·05% (23·28–40·49%)	43·54% (36·30–51·72%)
Lebanon	LBN	EMR	UMIC	89·58% (86·81–92·42%)	83·55% (77·24–90·04%)	77·58% (69·16–85·49%)	71·71% (60·62–83·02%)	62·17% (51·86–72·23%)	76·92% (69·14–84·64%)
Liberia	LBR	AFR	LIC	96·38% (94·01–98·14%)	96·09% (92·70–98·56%)	93·91% (88·04–97·60%)	92·21% (84·42–97·23%)	83·54% (73·68–91·25%)	92·43% (86·57–96·56%)
Libya	LBY	EMR	UMIC	85·30% (79·21–90·29%)	75·60% (65·79–83·90%)	67·52% (56·65–77·85%)	60·62% (47·80–72·07%)	53·33% (43·06–63·13%)	68·47% (58·50–77·45%)
St· Lucia	LCA	AMR	UMIC	65·19% (56·50–73·83%)	43·04% (29·55–57·67%)	33·66% (20·20–47·77%)	26·43% (14·79–40·71%)	25·99% (16·62–39·09%)	38·86% (27·53–51·81%)
Sri Lanka	LKA	SEAR	LMIC	80·61% (75·16–85·61%)	67·01% (57·73–75·82%)	58·06% (47·86–68·53%)	50·18% (38·44–61·08%)	44·78% (35·46–55·09%)	60·13% (50·93–69·23%)
Lesotho	LSO	AFR	LMIC	98·09% (96·23–99·24%)	98·52% (96·57–99·66%)	97·53% (93·99–99·43%)	96·89% (91·98–99·45%)	90·87% (82·95–96·43%)	96·38% (92·34–98·84%)
Morocco	MAR	EMR	LMIC	68·76% (63·87–73·87%)	48·23% (39·56–57·31%)	38·55% (29·49–48·39%)	32·58% (22·88–43·49%)	31·97% (23·71–41·30%)	44·02% (35·90–52·87%)
Moldova, Republic of	MDA	EUR	UMIC	77·01% (70·54–82·61%)	61·41% (51·49–71·27%)	51·48% (40·84–61·90%)	45·34% (34·47–56·95%)	42·17% (33·98–51·31%)	55·48% (46·26–64·81%)
Madagascar	MDG	AFR	LIC	87·61% (83·37–91·42%)	80·98% (73·55–87·52%)	74·34% (65·12–83·30%)	69·59% (58·30–80·27%)	60·81% (50·28–70·88%)	74·67% (66·12–82·68%)
Maldives	MDV	SEAR	UMIC	88·35% (83·06–92·87%)	81·03% (70·98–89·78%)	74·37% (62·22–84·98%)	67·73% (52·80–80·32%)	58·86% (46·65–71·42%)	74·07% (63·14–83·87%)

Country	Code	Region	Income level	Poorest	Poorer	Middle	Richer	Richest	Overall
Mexico	MEX	AMR	UMIC	75·37% (68·78–81·74%)	58·01% (46·91–68·88%)	48·32% (36·23–61·05%)	40·53% (28·67–52·83%)	37·44% (27·85–47·80%)	51·93% (41·69–62·46%)
Marshall Islands	MHL	WPR	UMIC	94·79% (91·86–97·43%)	93·71% (88·69–97·69%)	90·99% (84·14–96·54%)	88·56% (78·28–96·34%)	78·98% (67·12–89·23%)	89·41% (82·02–95·45%)
Macedonia, North	MKD	EUR	UMIC	86·04% (81·43–90·23%)	76·82% (67·84–84·97%)	69·28% (58·75–79·16%)	62·18% (48·93–73·86%)	54·24% (43·71–65·34%)	69·71% (60·13–78·71%)
Mali	MLI	AFR	LIC	79·37% (74·50–84·24%)	65·45% (56·37–73·83%)	56·00% (46·48–66·61%)	49·72% (39·04–60·25%)	45·27% (36·20–54·88%)	59·16% (50·52–67·96%)
Myanmar	MMR	SEAR	LMIC	82·39% (78·83–85·95%)	72·19% (65·06–78·58%)	64·18% (56·00–72·42%)	59·64% (49·66–68·87%)	53·03% (44·89–61·87%)	66·28% (58·89–73·54%)
Montenegro	MNE	EUR	UMIC	91·88% (86·34–96·12%)	87·48% (77·10–94·80%)	82·45% (69·43–92·16%)	76·86% (60·78–89·00%)	66·97% (52·96–80·00%)	81·13% (69·32–90·42%)
Mongolia	MNG	WPR	LMIC	80·47% (70·54–88·39%)	69·36% (54·83–80·98%)	60·94% (46·46–73·89%)	56·70% (40·75–71·17%)	51·17% (40·33–62·44%)	63·73% (50·58–75·38%)
Mozambique	MOZ	AFR	LIC	92·06% (87·96–95·48%)	89·51% (81·65–95·30%)	85·49% (75·23–93·37%)	82·92% (70·87–92·21%)	73·02% (60·75–83·56%)	84·60% (75·29–91·98%)
Mauritania	MRT	AFR	LMIC	77·41% (73·17–81·32%)	61·68% (54·78–68·78%)	52·30% (44·12–60·41%)	44·68% (35·89–53·97%)	40·65% (32·96–49·67%)	55·34% (48·18–62·83%)
Malawi	MWI	AFR	LIC	93·14% (90·11–95·61%)	90·66% (85·79–94·80%)	86·23% (78·42–92·29%)	82·83% (73·32–90·62%)	72·94% (63·42–81·59%)	85·16% (78·21–90·98%)
Malaysia	MYS	WPR	UMIC	64·25% (56·25–72·19%)	41·75% (28·84–56·30%)	32·42% (20·04–45·77%)	25·59% (14·57–39·24%)	25·53% (16·85–37·57%)	37·91% (27·31–50·21%)
Namibia	NAM	AFR	UMIC	86·42% (81·80–90·30%)	80·21% (73·56–85·86%)	73·78% (65·37–81·09%)	70·55% (60·19–80·15%)	62·07% (53·88–70·37%)	74·61% (66·96–81·56%)
Niger	NER	AFR	LIC	90·01% (86·54–93·21%)	85·05% (78·28–90·58%)	78·64% (69·46–86·55%)	74·38% (63·80–83·50%)	65·38% (56·55–74·38%)	78·69% (70·93–85·65%)
Nigeria	NGA	AFR	LMIC	81·87% (74·45–87·63%)	70·40% (59·85–79·24%)	61·44% (50·48–72·11%)	55·69% (42·36–67·30%)	50·08% (40·69–59·48%)	63·90% (53·56–73·15%)
Nicaragua	NIC	AMR	LMIC	90·91% (88·22–93·27%)	86·12% (80·96–91·45%)	80·59% (73·56–87·23%)	75·37% (65·48–84·53%)	65·53% (55·99–74·52%)	79·70% (72·84–86·20%)
Nepal	NPL	SEAR	LMIC	86·20% (81·70–89·70%)	78·36% (71·97–83·97%)	70·83% (62·42–78·36%)	65·74% (55·08–74·70%)	57·82% (49·83–66·47%)	71·79% (64·20–78·64%)
Pakistan	PAK	EMR	LMIC	78·21% (73·75–82·34%)	64·24% (57·41–71·02%)	55·06% (47·04–63·16%)	49·48% (39·76–59·17%)	45·15% (37·91–54·21%)	58·43% (51·17–65·98%)
Peru	PER	AMR	UMIC	70·25% (62·92–76·67%)	50·45% (40·06–61·70%)	40·53% (30·52–51·69%)	34·60% (24·15–46·75%)	33·78% (25·57–43·33%)	45·92% (36·64–56·03%)
Philippines	PHL	WPR	LMIC	78·78% (72·60–84·06%)	67·01% (58·61–74·06%)	59·00% (50·49–67·09%)	55·03% (44·26–65·59%)	49·51% (41·40–57·97%)	61·86% (53·47–69·76%)

Country	Code	Region	Income level	Poorest	Poorer	Middle	Richer	Richest	Overall
Papua New Guinea	PNG	WPR	LMIC	64·69% (59·75–69·74%)	44·13% (34·45–54·88%)	35·47% (26·25–45·46%)	30·52% (20·69–42·36%)	30·19% (21·51–40·52%)	41·00% (32·53–50·59%)
Korea, Democratic People's Republic of	PRK	SEAR	LIC	89·99% (86·75–92·80%)	86·35% (79·74–92·03%)	81·68% (72·83–89·14%)	78·82% (67·95–88·34%)	69·02% (57·61–79·12%)	81·17% (72·97–88·29%)
Paraguay	PRY	AMR	UMIC	80·77% (77·83–83·78%)	67·56% (61·18–74·07%)	58·52% (51·00–66·40%)	51·52% (41·91–61·16%)	46·16% (38·63–55·09%)	60·91% (54·11–68·10%)
West Bank and Gaza	PSE	EMR	LMIC	75·00% (69·59–80·43%)	57·73% (46·83–68·73%)	48·35% (37·16–60·52%)	40·64% (28·60–53·48%)	37·32% (27·43–48·95%)	51·81% (41·92–62·42%)
Russian Federation	RUS	EUR	UMIC	62·29% (53·72–70·46%)	38·89% (27·02–52·30%)	29·57% (18·94–42·57%)	24·17% (13·87–37·39%)	25·37% (16·51–36·21%)	36·06% (26·01–47·79%)
Rwanda	RWA	AFR	LIC	87·19% (82·89–90·98%)	79·53% (71·52–86·73%)	72·47% (62·22–81·71%)	66·69% (55·48–77·28%)	58·22% (47·15–68·39%)	72·82% (63·85–81·02%)
Sudan	SDN	EMR	LIC	85·78% (81·90–89·60%)	77·03% (69·96–83·52%)	69·12% (60·14–77·90%)	63·40% (52·83–73·19%)	55·86% (47·28–65·02%)	70·24% (62·42–77·85%)
Senegal	SEN	AFR	LMIC	79·60% (76·28–83·03%)	65·80% (59·60–71·99%)	56·81% (49·23–64·68%)	49·84% (41·02–59·46%)	44·77% (36·64–53·38%)	59·36% (52·55–66·51%)
Solomon Islands	SLB	WPR	LMIC	80·88% (77·12–84·72%)	67·75% (59·96–75·53%)	59·02% (49·87–68·25%)	51·61% (41·50–62·03%)	45·90% (36·73–55·59%)	61·03% (53·03–69·22%)
Sierra Leone	SLE	AFR	LIC	95·55% (93·06–97·43%)	94·97% (90·66–97·98%)	92·36% (85·97–96·75%)	90·66% (82·46–96·32%)	81·62% (71·57–89·54%)	91·03% (84·74–95·61%)
El Salvador	SLV	AMR	LMIC	87·35% (83·65–90·77%)	79·39% (72·56–85·80%)	72·23% (63·60–80·11%)	65·69% (54·81–75·86%)	57·22% (47·65–66·82%)	72·37% (64·45–79·87%)
Somalia	SOM	EMR	LIC	92·55% (88·49–95·86%)	90·26% (83·49–95·47%)	85·30% (75·28–92·84%)	82·93% (72·04–91·62%)	73·88% (65·03–83·49%)	84·98% (76·86–91·86%)
Serbia	SRB	EUR	UMIC	94·12% (89·34–97·54%)	91·54% (83·37–97·03%)	87·73% (76·51–95·37%)	83·30% (68·88–93·57%)	73·28% (59·47–85·15%)	85·99% (75·51–93·73%)
South Sudan	SSD	AFR	LIC	87·46% (84·65–90·14%)	80·80% (75·35–85·72%)	74·08% (66·54–81·29%)	69·50% (60·08–78·20%)	60·81% (52·02–69·22%)	74·53% (67·73–80·91%)
São Tomé and Principe	STP	AFR	LMIC	90·44% (86·03–93·82%)	85·52% (79·02–90·87%)	79·44% (70·91–86·66%)	74·47% (62·89–83·49%)	65·12% (55·78–74·15%)	79·00% (70·92–85·80%)
Suriname	SUR	AMR	UMIC	90·51% (87·77–92·89%)	85·86% (81·40–89·80%)	79·57% (73·19–85·35%)	75·16% (66·38–83·87%)	66·09% (58·92–74·43%)	79·44% (73·53–85·27%)
Swaziland	SWZ	AFR	LMIC	84·05% (80·37–87·41%)	75·13% (69·21–80·64%)	67·48% (60·35–74·20%)	62·98% (53·43–71·90%)	55·67% (48·19–63·16%)	69·06% (62·31–75·46%)
Syrian Arab Republic	SYR	EMR	LIC	79·80% (72·49–86·69%)	66·30% (53·30–78·06%)	57·38% (43·82–71·35%)	50·88% (37·40–64·97%)	45·81% (34·38–57·69%)	60·03% (48·28–71·75%)
Chad	TCD	AFR	LIC	81·99% (74·71–88·78%)	70·92% (57·22–83·21%)	61·86% (47·36–75·95%)	57·08% (41·80–72·15%)	51·71% (40·46–64·81%)	64·71% (52·31–76·98%)

Country	Code	Region	Income level	Poorest	Poorer	Middle	Richer	Richest	Overall
Togo	TGO	AFR	LIC	84·70% (79·99–89·01%)	74·94% (65·70–83·13%)	67·11% (56·19–77·34%)	60·86% (49·08–72·45%)	53·47% (42·69–63·73%)	68·22% (58·73–77·13%)
Thailand	THA	SEAR	UMIC	60·70% (54·11–66·87%)	37·17% (27·39–49·21%)	28·20% (18·78–39·10%)	22·67% (13·84–33·51%)	23·81% (15·85–33·66%)	34·51% (25·99–44·47%)
Tajikistan	TJK	EUR	LMIC	90·73% (87·25–93·95%)	86·52% (80·16–91·88%)	80·20% (71·28–88·06%)	76·45% (66·18–85·48%)	67·60% (60·22–76·60%)	80·30% (73·02–87·19%)
Turkmenistan	TKM	EUR	UMIC	84·18% (78·94–88·64%)	73·37% (62·53–82·57%)	65·47% (53·30–76·88%)	57·89% (42·99–71·14%)	50·65% (39·46–62·81%)	66·31% (55·44–76·41%)
Timor-Leste	TLS	SEAR	LMIC	90·85% (87·57–93·61%)	87·56% (82·68–92·32%)	82·94% (76·28–89·31%)	79·80% (69·57–88·76%)	69·90% (60·07–79·80%)	82·21% (75·23–88·76%)
Tonga	TON	WPR	UMIC	76·13% (71·12–80·97%)	59·47% (49·24–69·70%)	50·13% (38·33–61·52%)	42·25% (30·74–54·77%)	38·54% (29·04–49·91%)	53·31% (43·69–63·37%)
Tunisia	TUN	EMR	LMIC	87·03% (83·66–90·16%)	78·80% (72·48–85·09%)	71·56% (63·80–79·16%)	64·93% (54·18–75·02%)	56·57% (47·24–66·02%)	71·78% (64·27–79·09%)
Turkey	TUR	EUR	UMIC	65·94% (59·73–72·34%)	43·63% (33·63–54·80%)	33·97% (23·95–45·15%)	27·05% (17·76–38·07%)	26·98% (19·46–36·69%)	39·51% (30·90–49·41%)
Tuvalu	TUV	WPR	UMIC	89·17% (86·21–92·19%)	83·84% (76·44–90·65%)	78·38% (68·97–87·23%)	73·89% (61·40–86·15%)	64·27% (53·02–75·80%)	77·91% (69·21–86·41%)
Tanzania, United Republic of	TZA	AFR	LMIC	81·20% (77·81–84·61%)	69·30% (62·64–75·54%)	60·90% (52·71–69·02%)	54·98% (45·61–64·56%)	48·97% (40·17–57·79%)	63·07% (55·79–70·30%)
Uganda	UGA	AFR	LIC	79·91% (75·72–84·12%)	67·08% (58·60–74·83%)	58·35% (49·08–68·13%)	52·62% (42·33–63·32%)	47·32% (38·44–56·76%)	61·06% (52·83–69·43%)
Ukraine	UKR	EUR	LMIC	82·76% (77·37–87·17%)	71·63% (63·12–79·01%)	62·55% (53·49–71·43%)	56·74% (46·28–67·43%)	50·98% (43·38–58·71%)	64·93% (56·73–72·75%)
Uzbekistan	UZB	EUR	LMIC	83·66% (78·42–88·10%)	73·38% (64·87–81·37%)	64·40% (54·02–73·71%)	58·95% (48·52–69·69%)	52·91% (45·07–62·35%)	66·66% (58·18–75·05%)
St· Vincent and the Grenadines	VCT	AMR	UMIC	70·39% (62·76–77·80%)	50·49% (36·93–64·36%)	40·97% (27·06–55·10%)	33·24% (20·42–48·07%)	31·39% (21·13–44·36%)	45·30% (33·66–57·94%)
Venezuela	VEN	AMR	UMIC	70·36% (61·36–79·16%)	50·28% (35·46–65·83%)	40·69% (26·35–56·03%)	32·87% (19·55–48·42%)	31·13% (20·83–43·68%)	45·07% (32·71–58·63%)
Vietnam	VNM	WPR	LMIC	74·64% (67·36–81·50%)	58·04% (46·74–69·75%)	48·28% (36·75–59·88%)	42·86% (30·91–56·48%)	40·42% (31·39–50·81%)	52·85% (42·63–63·68%)
Vanuatu	VUT	WPR	LMIC	71·22% (66·37–76·46%)	51·57% (42·62–60·76%)	41·85% (32·30–51·90%)	34·26% (24·92–44·63%)	32·47% (24·54–42·39%)	46·27% (38·15–55·23%)
Samoa	WSM	WPR	LMIC	78·90% (73·99–83·65%)	64·27% (53·83–74·69%)	55·35% (44·10–67·07%)	47·66% (34·91–60·83%)	42·71% (32·38–54·36%)	57·78% (47·84–68·12%)
Kosovo	XKX	EUR	UMIC	86·18% (82·30–89·83%)	77·16% (69·49–84·16%)	69·62% (60·61–78·50%)	62·70% (50·74–73·39%)	54·73% (45·17–64·59%)	70·08% (61·66–78·09%)

Country	Code	Region	Income level	Poorest	Poorer	Middle	Richer	Richest	Overall
Yemen	YEM	EMR	LIC	83·39% (78·91–87·78%)	72·54% (64·79–79·69%)	63·91% (54·52–73·45%)	57·61% (47·32–67·53%)	51·21% (42·67–60·50%)	65·73% (57·64–73·79%)
South Africa	ZAF	AFR	UMIC	89·18% (85·17–92·67%)	85·04% (78·81–90·47%)	79·94% (71·90–87·22%)	76·92% (65·56–87·07%)	67·37% (56·68–77·47%)	79·69% (71·62–86·98%)
Zambia	ZMB	AFR	LIC	80·05% (75·31–85·03%)	68·33% (58·35–77·19%)	60·01% (49·45–70·25%)	55·69% (44·40–67·34%)	50·09% (40·55–60·28%)	62·83% (53·61–72·02%)
Zimbabwe	ZWE	AFR	LMIC	92·65% (90·25–94·73%)	89·81% (85·65–93·78%)	85·28% (79·09–90·94%)	81·59% (72·93–89·48%)	71·57% (62·37–79·73%)	84·18% (78·06–89·73%)

* All countries include 135 low- and middle-income countries analyzed. Values in parentheses represent equal-tailed 95% credible intervals.

Table S13. Predicted proportion of households experiencing catastrophic direct medical costs due to tuberculosis, stratified by income quintile*.

Country	Code	Region	Income level	Poorest	Poorer	Middle	Richer	Richest	Overall
Afghanistan	AFG	EMR	LIC	62·22% (47·80–76·33%)	42·12% (21·66–64·21%)	31·86% (16·95–50·26%)	25·69% (12·57–42·82%)	26·53% (14·26–41·65%)	37·69% (22·65–55·05%)
Angola	AGO	AFR	LMIC	25·73% (17·95–34·50%)	7·85% (3·05–16·55%)	5·48% (2·44–9·70%)	3·78% (1·59–6·85%)	5·01% (2·66–8·35%)	9·57% (5·54–15·19%)
Albania	ALB	EUR	UMIC	24·71% (16·55–33·33%)	7·21% (2·81–14·33%)	5·08% (2·29–9·35%)	3·47% (1·51–6·63%)	4·68% (2·33–8·11%)	9·03% (5·10–14·35%)
Argentina	ARG	AMR	UMIC	41·46% (28·89–54·91%)	19·45% (7·85–35·38%)	13·88% (6·08–24·86%)	10·31% (4·15–19·89%)	11·90% (5·88–20·07%)	19·40% (10·57–31·02%)
Armenia	ARM	EUR	UMIC	51·02% (38·21–63·99%)	28·75% (13·97–46·82%)	20·96% (10·63–34·41%)	16·15% (7·67–28·38%)	17·62% (9·57–27·68%)	26·90% (16·01–40·26%)
American Samoa	ASM	WPR	UMIC	23·59% (10·94–39·74%)	6·99% (1·20–19·57%)	4·97% (1·03–12·71%)	3·43% (0·65–9·44%)	4·57% (1·02–10·84%)	8·71% (2·97–18·46%)
Azerbaijan	AZE	EUR	UMIC	27·11% (13·85–42·85%)	9·06% (1·91–23·15%)	6·33% (1·51–14·76%)	4·46% (1·04–10·93%)	5·70% (1·67–12·36%)	10·53% (4·00–20·81%)
Burundi	BDI	AFR	LIC	62·42% (51·43–73·21%)	42·18% (25·33–60·30%)	31·80% (19·50–46·26%)	25·57% (15·21–38·99%)	26·44% (16·63–37·74%)	37·68% (25·62–51·30%)
Benin	BEN	AFR	LMIC	27·64% (16·89–40·08%)	9·14% (2·60–20·92%)	6·39% (2·27–13·03%)	4·47% (1·42–9·70%)	5·75% (2·32–10·74%)	10·68% (5·10–18·90%)
Burkina Faso	BFA	AFR	LIC	37·72% (29·19–46·59%)	16·02% (7·81–27·06%)	11·34% (6·10–18·13%)	8·22% (4·40–13·64%)	9·84% (6·14–14·78%)	16·63% (10·73–24·04%)
Bangladesh	BGD	SEAR	LMIC	16·99% (11·52–23·72%)	3·59% (1·19–8·00%)	2·53% (1·06–4·63%)	1·66% (0·65–3·22%)	2·49% (1·31–4·13%)	5·45% (3·15–8·74%)
Bulgaria	BGR	EUR	UMIC	27·03% (18·63–36·71%)	8·63% (3·09–17·56%)	6·03% (2·49–11·22%)	4·20% (1·66–8·25%)	5·49% (2·73–9·43%)	10·27% (5·72–16·64%)
Bosnia and Herzegovina	BIH	EUR	UMIC	42·11% (20·65–64·64%)	20·80% (4·40–46·84%)	15·12% (3·51–34·57%)	11·39% (2·24–28·84%)	12·92% (3·22–29·60%)	20·47% (6·80–40·90%)
Belarus	BLR	EUR	UMIC	31·65% (18·90–44·77%)	11·80% (3·66–25·83%)	8·29% (2·73–16·28%)	5·92% (1·82–12·47%)	7·33% (3·03–14·23%)	13·00% (6·03–22·72%)
Belize	BLZ	AMR	UMIC	18·36% (12·18–25·65%)	4·15% (1·42–8·91%)	2·93% (1·17–5·54%)	1·94% (0·76–3·72%)	2·84% (1·33–4·98%)	6·05% (3·37–9·76%)
Bolivia	BOL	AMR	LMIC	39·08% (26·55–50·86%)	17·26% (7·13–31·42%)	12·31% (5·61–21·67%)	9·01% (3·98–16·57%)	10·63% (5·09–18·47%)	17·66% (9·67–27·80%)
Brazil	BRA	AMR	UMIC	32·52% (20·06–47·41%)	12·35% (3·89–26·17%)	8·76% (3·23–17·97%)	6·25% (2·12–13·33%)	7·72% (2·99–14·99%)	13·52% (6·46–23·97%)
Bhutan	BTN	SEAR	LMIC	25·33% (11·59–42·22%)	8·10% (1·46–21·91%)	5·64% (1·23–14·33%)	3·95% (0·75–10·56%)	5·12% (1·40–11·89%)	9·63% (3·29–20·18%)

Country	Code	Region	Income level	Poorest	Poorer	Middle	Richer	Richest	Overall
Botswana	BWA	AFR	UMIC	31·01% (21·00–42·32%)	11·12% (4·26–21·14%)	7·85% (3·42–13·94%)	5·55% (2·29–10·47%)	6·99% (3·28–12·15%)	12·51% (6·85–20·01%)
Central African Republic	CAF	AFR	LIC	51·43% (34·06–69·41%)	29·57% (10·69–53·96%)	21·75% (8·30–41·08%)	16·88% (6·11–34·14%)	18·27% (7·85–32·51%)	27·58% (13·40–46·22%)
China	CHN	WPR	UMIC	20·72% (9·59–34·73%)	5·57% (0·96–16·04%)	3·87% (0·80–10·04%)	2·64% (0·50–7·53%)	3·64% (0·96–8·61%)	7·29% (2·56–15·39%)
Côte d'Ivoire	CIV	AFR	LMIC	30·67% (16·12–47·72%)	11·39% (2·53–27·57%)	7·99% (2·03–18·44%)	5·73% (1·36–14·51%)	7·07% (2·16–15·31%)	12·57% (4·84–24·71%)
Cameroon	CMR	AFR	LMIC	32·16% (23·57–40·66%)	11·82% (5·26–21·24%)	8·34% (4·16–13·83%)	5·90% (2·92–10·09%)	7·38% (4·25–11·45%)	13·12% (8·03–19·46%)
Congo, Democratic Republic of the	COD	AFR	LIC	29·73% (21·51–39·72%)	10·29% (4·05–19·97%)	7·20% (3·57–12·54%)	5·05% (2·25–9·22%)	6·43% (3·63–10·13%)	11·74% (7·00–18·32%)
Congo	COG	AFR	LMIC	21·22% (14·16–29·39%)	5·48% (1·86–11·80%)	3·82% (1·64–7·07%)	2·58% (0·98–4·88%)	3·61% (1·77–6·24%)	7·34% (4·08–11·88%)
Colombia	COL	AMR	UMIC	31·76% (19·96–44·79%)	11·72% (3·95–23·69%)	8·30% (3·13–16·41%)	5·89% (2·13–11·79%)	7·36% (2·94–13·83%)	13·01% (6·42–22·10%)
Comoros	COM	AFR	LMIC	45·28% (26·47–64·72%)	23·37% (7·71–46·03%)	16·97% (5·35–33·81%)	12·86% (3·87–28·05%)	14·41% (5·10–28·83%)	22·58% (9·70–40·29%)
Cabo Verde	CPV	AFR	LMIC	19·21% (12·87–26·23%)	4·52% (1·56–9·60%)	3·18% (1·32–6·11%)	2·11% (0·81–4·14%)	3·06% (1·55–5·23%)	6·42% (3·62–10·26%)
Costa Rica	CRI	AMR	UMIC	37·53% (16·51–61·82%)	16·86% (3·10–41·38%)	12·17% (2·38–30·41%)	9·02% (1·67–24·65%)	10·52% (2·30–26·20%)	17·22% (5·19–36·89%)
Cuba	CUB	AMR	UMIC	28·55% (15·73–43·76%)	9·77% (2·48–22·91%)	6·92% (2·05–15·68%)	4·87% (1·31–11·52%)	6·20% (2·03–13·10%)	11·26% (4·72–21·40%)
Djibouti	DJI	EMR	LMIC	13·82% (7·55–22·59%)	2·56% (0·50–6·86%)	1·79% (0·50–4·25%)	1·16% (0·30–2·88%)	1·82% (0·65–3·85%)	4·23% (1·90–8·09%)
Dominica	DMA	AMR	UMIC	33·81% (16·80–54·85%)	13·64% (2·96–32·31%)	9·75% (2·27–22·51%)	7·07% (1·63–17·91%)	8·54% (2·34–19·59%)	14·56% (5·20–29·43%)
Dominican Republic	DOM	AMR	UMIC	31·66% (20·63–43·47%)	11·68% (4·20–23·73%)	8·21% (3·34–15·17%)	5·84% (2·21–11·34%)	7·27% (3·35–13·03%)	12·93% (6·74–21·35%)
Algeria	DZA	AFR	LMIC	28·40% (21·24–36·29%)	9·34% (4·01–17·19%)	6·56% (3·37–10·92%)	4·56% (2·21–7·98%)	5·92% (3·41–9·21%)	10·96% (6·85–16·32%)
Ecuador	ECU	AMR	UMIC	36·43% (27·55–46·50%)	15·02% (6·70–26·30%)	10·64% (5·62–17·69%)	7·69% (3·69–13·64%)	9·27% (5·08–14·64%)	15·81% (9·73–23·76%)
Egypt	EGY	EMR	LMIC	25·89% (18·30–34·59%)	7·86% (3·14–15·51%)	5·54% (2·53–9·81%)	3·81% (1·71–6·94%)	5·06% (2·70–8·47%)	9·63% (5·67–15·06%)
Eritrea	ERI	AFR	LIC	39·32% (30·76–48·27%)	17·34% (8·51–29·15%)	12·30% (6·83–19·24%)	8·98% (4·74–14·34%)	10·60% (6·66–15·29%)	17·71% (11·50–25·26%)

Country	Code	Region	Income level	Poorest	Poorer	Middle	Richer	Richest	Overall
Ethiopia	ETH	AFR	LIC	24·43% (17·34–33·59%)	7·12% (2·59–14·51%)	4·97% (2·32–9·18%)	3·40% (1·38–6·27%)	4·58% (2·46–7·45%)	8·90% (5·22–14·20%)
Fiji	FJI	WPR	UMIC	16·42% (10·74–23·03%)	3·36% (1·13–7·50%)	2·39% (0·93–4·55%)	1·56% (0·59–2·97%)	2·37% (1·12–4·21%)	5·22% (2·90–8·45%)
Micronesia	FSM	WPR	LMIC	32·75% (17·07–52·24%)	12·86% (2·97–31·65%)	9·18% (2·23–21·97%)	6·60% (1·38–16·46%)	8·04% (2·21–17·96%)	13·89% (5·17–28·06%)
Gabon	GAB	AFR	UMIC	19·62% (11·74–29·29%)	4·78% (1·36–11·15%)	3·36% (1·15–7·02%)	2·25% (0·65–4·86%)	3·22% (1·27–6·20%)	6·65% (3·23–11·70%)
Georgia	GEO	EUR	UMIC	38·34% (25·92–50·93%)	16·78% (6·65–32·37%)	11·90% (4·99–21·35%)	8·73% (3·43–16·33%)	10·28% (5·11–17·74%)	17·21% (9·22–27·75%)
Ghana	GHA	AFR	LMIC	35·57% (23·78–47·78%)	14·49% (5·49–26·85%)	10·27% (4·58–18·51%)	7·41% (3·02–14·07%)	8·97% (4·20–15·66%)	15·34% (8·21–24·57%)
Guinea	GIN	AFR	LIC	32·40% (19·98–46·13%)	12·34% (3·78–26·69%)	8·65% (3·06–17·34%)	6·20% (2·04–12·85%)	7·62% (3·27–14·31%)	13·44% (6·43–23·46%)
Gambia	GMB	AFR	LIC	36·64% (23·64–50·87%)	15·52% (5·08–31·52%)	10·96% (4·20–20·79%)	7·99% (2·80–15·74%)	9·49% (4·34–17·21%)	16·12% (8·01–27·23%)
Guinea-Bissau	GNB	AFR	LIC	60·55% (48·95–71·50%)	39·78% (23·14–57·82%)	29·81% (18·16–43·48%)	23·81% (13·94–37·11%)	24·81% (15·29–35·94%)	35·75% (23·90–49·17%)
Equatorial Guinea	GNQ	AFR	UMIC	30·23% (16·44–46·20%)	11·02% (2·44–25·85%)	7·74% (2·00–17·02%)	5·52% (1·28–13·35%)	6·88% (2·40–14·13%)	12·28% (4·91–23·31%)
Grenada	GRD	AMR	UMIC	30·59% (15·36–49·72%)	11·24% (2·45–26·76%)	8·01% (1·97–18·90%)	5·71% (1·31–14·39%)	7·11% (2·02–16·33%)	12·53% (4·62–25·22%)
Guatemala	GTM	AMR	UMIC	29·86% (20·47–39·77%)	10·32% (4·24–19·80%)	7·29% (3·42–13·10%)	5·11% (2·20–9·51%)	6·52% (3·31–11·32%)	11·82% (6·73–18·70%)
Guyana	GUY	AMR	UMIC	20·42% (13·31–28·23%)	5·04% (1·77–10·87%)	3·56% (1·49–6·91%)	2·38% (0·95–4·64%)	3·39% (1·61–6·03%)	6·96% (3·83–11·34%)
Honduras	HND	AMR	LMIC	40·41% (30·43–50·87%)	18·37% (8·30–31·73%)	13·05% (6·51–21·20%)	9·61% (4·59–16·55%)	11·21% (6·51–17·48%)	18·53% (11·27–27·57%)
Haiti	HTI	AMR	LMIC	32·27% (25·49–39·40%)	11·87% (5·69–21·07%)	8·35% (4·70–12·95%)	5·91% (3·19–9·51%)	7·39% (4·69–10·57%)	13·16% (8·75–18·70%)
Indonesia	IDN	SEAR	LMIC	22·18% (15·25–29·69%)	5·88% (2·22–11·78%)	4·13% (1·91–7·29%)	2·79% (1·16–5·14%)	3·88% (1·96–6·33%)	7·77% (4·50–12·05%)
India	IND	SEAR	LMIC	23·44% (15·81–32·46%)	6·62% (2·20–13·87%)	4·61% (1·96–8·61%)	3·15% (1·21–5·99%)	4·28% (2·15–7·24%)	8·42% (4·67–13·64%)
Iran	IRN	EMR	LMIC	32·69% (19·68–46·62%)	12·46% (3·91–26·08%)	8·85% (3·17–18·19%)	6·32% (1·97–13·49%)	7·80% (2·88–15·37%)	13·63% (6·32–23·95%)
Iraq	IRQ	EMR	UMIC	34·54% (19·70–51·02%)	14·03% (3·94–30·95%)	9·90% (3·10–19·80%)	7·18% (2·03–15·65%)	8·64% (3·13–16·65%)	14·86% (6·38–26·81%)

Country	Code	Region	Income level	Poorest	Poorer	Middle	Richer	Richest	Overall
Jamaica	JAM	AMR	UMIC	18·34% (11·19–27·00%)	4·18% (1·26–9·45%)	2·96% (1·06–6·11%)	1·96% (0·66–4·01%)	2·86% (1·19–5·48%)	6·06% (3·07–10·41%)
Jordan	JOR	EMR	UMIC	40·55% (26·42–54·64%)	18·64% (7·15–35·17%)	13·34% (5·55–24·63%)	9·85% (3·78–19·00%)	11·46% (4·88–20·88%)	18·77% (9·56–30·86%)
Kazakhstan	KAZ	EUR	UMIC	16·83% (5·90–32·26%)	3·99% (0·35–13·14%)	2·76% (0·30–8·73%)	1·86% (0·22–6·38%)	2·66% (0·44–7·36%)	5·62% (1·44–13·57%)
Kenya	KEN	AFR	LMIC	32·46% (24·73–41·27%)	12·02% (5·48–21·12%)	8·48% (4·38–13·77%)	6·00% (3·05–10·04%)	7·50% (4·30–11·36%)	13·29% (8·39–19·51%)
Kyrgyz Republic	KGZ	EUR	LMIC	36·28% (20·79–53·29%)	15·46% (4·22–34·17%)	10·93% (3·35–23·04%)	7·99% (2·28–18·03%)	9·47% (3·45–19·07%)	16·03% (6·82–29·52%)
Cambodia	KHM	WPR	LMIC	35·51% (25·35–47·18%)	14·38% (5·83–26·74%)	10·19% (4·66–18·06%)	7·33% (3·29–13·46%)	8·89% (4·50–15·05%)	15·26% (8·73–24·10%)
Kiribati	KIR	WPR	LMIC	43·17% (28·41–59·24%)	21·20% (8·02–41·56%)	15·24% (6·03–29·13%)	11·42% (4·11–23·68%)	12·97% (5·72–23·90%)	20·80% (10·46–35·50%)
Lao People's Democratic Republic	LAO	WPR	LMIC	16·99% (11·72–23·55%)	3·60% (1·19–8·39%)	2·53% (1·10–4·82%)	1·66% (0·62–3·22%)	2·49% (1·29–4·12%)	5·46% (3·18–8·82%)
Lebanon	LBN	EMR	UMIC	31·73% (18·43–47·17%)	11·90% (3·36–26·83%)	8·45% (2·62–18·55%)	6·02% (1·73–13·75%)	7·46% (2·53–15·38%)	13·11% (5·73–24·34%)
Liberia	LBR	AFR	LIC	52·96% (35·45–69·88%)	31·21% (11·69–54·72%)	23·03% (9·49–41·26%)	17·95% (6·73–34·94%)	19·32% (8·17–34·42%)	28·90% (14·31–47·04%)
Libya	LBY	EMR	UMIC	34·48% (23·05–46·66%)	13·63% (5·26–26·11%)	9·66% (4·14–17·46%)	6·94% (2·95–13·26%)	8·48% (4·05–15·15%)	14·64% (7·89–23·73%)
St· Lucia	LCA	AMR	UMIC	11·22% (7·11–16·35%)	1·66% (0·47–3·96%)	1·20% (0·42–2·44%)	0·76% (0·25–1·53%)	1·28% (0·59–2·37%)	3·22% (1·77–5·33%)
Sri Lanka	LKA	SEAR	LMIC	24·73% (15·21–35·13%)	7·29% (2·51–15·74%)	5·14% (1·90–10·33%)	3·53% (1·26–7·24%)	4·73% (2·06–9·07%)	9·08% (4·59–15·50%)
Lesotho	LSO	AFR	LMIC	61·88% (41·78–80·38%)	42·13% (16·26–70·42%)	32·07% (12·52–55·99%)	25·98% (9·81–50·38%)	26·77% (11·10–47·12%)	37·77% (18·29–60·86%)
Morocco	MAR	EMR	LMIC	22·13% (13·30–32·46%)	6·03% (1·70–14·12%)	4·20% (1·42–8·64%)	2·86% (0·91–6·08%)	3·92% (1·66–7·59%)	7·83% (3·80–13·78%)
Moldova, Republic of	MDA	EUR	UMIC	34·87% (21·91–47·90%)	14·11% (4·85–29·12%)	9·95% (3·66–18·69%)	7·20% (2·45–14·05%)	8·69% (3·84–15·93%)	14·96% (7·34–25·14%)
Madagascar	MDG	AFR	LIC	35·45% (26·14–45·44%)	14·28% (6·41–26·00%)	10·10% (4·93–16·85%)	7·25% (3·47–12·62%)	8·81% (5·02–14·12%)	15·18% (9·19–23·01%)
Maldives	MDV	SEAR	UMIC	32·20% (18·21–47·54%)	12·21% (3·33–26·71%)	8·69% (2·73–18·71%)	6·21% (1·74–14·16%)	7·67% (2·51–15·93%)	13·39% (5·70–24·61%)
Mexico	MEX	AMR	UMIC	22·40% (15·18–30·43%)	5·99% (2·18–12·09%)	4·21% (1·90–7·57%)	2·85% (1·27–5·24%)	3·96% (2·01–6·70%)	7·88% (4·51–12·41%)

Country	Code	Region	Income level	Poorest	Poorer	Middle	Richer	Richest	Overall
Marshall Islands	MHL	WPR	UMIC	37·31% (16·35–62·88%)	16·88% (2·34–44·06%)	12·20% (2·02–31·89%)	9·07% (1·32–26·73%)	10·53% (2·08–25·68%)	17·20% (4·82–38·25%)
Macedonia, North	MKD	EUR	UMIC	29·39% (17·70–41·52%)	10·15% (3·01–21·12%)	7·19% (2·60–14·57%)	5·06% (1·66–10·77%)	6·44% (2·46–12·67%)	11·65% (5·49–20·13%)
Mali	MLI	AFR	LIC	33·42% (23·91–44·10%)	12·85% (5·08–24·02%)	9·03% (4·27–15·75%)	6·44% (2·86–11·39%)	7·93% (4·41–12·61%)	13·93% (8·10–21·58%)
Myanmar	MMR	SEAR	LMIC	33·04% (25·56–41·13%)	12·47% (5·74–22·15%)	8·76% (4·65–13·92%)	6·23% (3·10–10·45%)	7·73% (4·73–11·44%)	13·65% (8·76–19·82%)
Montenegro	MNE	EUR	UMIC	37·31% (17·98–58·62%)	16·51% (3·38–38·60%)	11·89% (2·60–28·70%)	8·78% (1·79–23·02%)	10·29% (2·56–24·20%)	16·96% (5·66–34·63%)
Mongolia	MNG	WPR	LMIC	36·98% (24·35–51·18%)	15·74% (5·57–31·63%)	11·16% (4·35–21·36%)	8·15% (2·73–16·41%)	9·70% (4·14–17·57%)	16·35% (8·23–27·63%)
Mozambique	MOZ	AFR	LIC	37·25% (23·26–54·74%)	16·15% (4·85–35·50%)	11·52% (3·98–23·95%)	8·41% (2·55–18·99%)	9·94% (3·84–19·48%)	16·66% (7·70–30·53%)
Mauritania	MRT	AFR	LMIC	22·51% (14·74–31·21%)	6·07% (2·13–12·66%)	4·27% (1·76–8·01%)	2·89% (1·13–5·43%)	4·00% (1·94–6·97%)	7·95% (4·34–12·86%)
Malawi	MWI	AFR	LIC	47·78% (36·46–59·56%)	25·26% (12·44–41·52%)	18·27% (9·57–29·47%)	13·85% (7·01–23·15%)	15·43% (8·75–24·27%)	24·12% (14·84–35·60%)
Malaysia	MYS	WPR	UMIC	12·00% (8·03–16·70%)	1·87% (0·57–4·39%)	1·34% (0·53–2·61%)	0·85% (0·30–1·66%)	1·42% (0·72–2·43%)	3·50% (2·03–5·56%)
Namibia	NAM	AFR	UMIC	37·36% (25·22–51·02%)	15·97% (6·25–30·75%)	11·32% (4·80–20·25%)	8·27% (3·24–15·93%)	9·82% (4·67–17·12%)	16·55% (8·84–27·01%)
Niger	NER	AFR	LIC	48·72% (40·01–57·45%)	26·10% (14·51–40·36%)	18·85% (11·72–27·21%)	14·32% (8·23–21·58%)	15·89% (10·62–21·78%)	24·77% (17·02–33·68%)
Nigeria	NGA	AFR	LMIC	37·79% (26·43–49·27%)	16·21% (6·80–29·52%)	11·49% (5·27–19·45%)	8·38% (3·50–15·02%)	9·96% (5·15–16·24%)	16·76% (9·43–25·90%)
Nicaragua	NIC	AMR	LMIC	37·29% (23·91–51·84%)	15·96% (5·49–31·39%)	11·38% (4·34–22·13%)	8·29% (2·87–16·92%)	9·86% (4·05–18·52%)	16·56% (8·13–28·16%)
Nepal	NPL	SEAR	LMIC	38·55% (29·77–47·51%)	16·67% (8·16–28·49%)	11·82% (6·55–18·53%)	8·60% (4·53–13·77%)	10·23% (6·23–15·27%)	17·18% (11·05–24·72%)
Pakistan	PAK	EMR	LMIC	30·73% (22·76–39·18%)	10·87% (4·66–19·88%)	7·63% (3·94–12·17%)	5·37% (2·50–8·85%)	6·79% (4·00–10·33%)	12·28% (7·57–18·08%)
Peru	PER	AMR	UMIC	27·34% (16·12–40·12%)	8·98% (2·67–20·21%)	6·27% (2·11–12·70%)	4·39% (1·38–9·72%)	5·67% (2·23–11·10%)	10·53% (4·90–18·77%)
Philippines	PHL	WPR	LMIC	27·72% (18·69–37·98%)	9·02% (3·37–18·16%)	6·33% (2·69–11·70%)	4·40% (1·75–8·38%)	5·73% (2·83–9·90%)	10·64% (5·87–17·22%)
Papua New Guinea	PNG	WPR	LMIC	11·94% (7·13–18·92%)	1·90% (0·51–5·05%)	1·35% (0·44–3·04%)	0·87% (0·25–1·99%)	1·42% (0·58–2·72%)	3·50% (1·78–6·34%)

Country	Code	Region	Income level	Poorest	Poorer	Middle	Richer	Richest	Overall
Korea, Democratic People's Republic of	PRK	SEAR	LIC	31·43% (19·19–47·59%)	11·67% (3·38–26·66%)	8·26% (2·76–17·44%)	5·87% (1·83–13·44%)	7·29% (2·75–14·51%)	12·90% (5·98–23·93%)
Paraguay	PRY	AMR	UMIC	28·74% (21·43–36·70%)	9·57% (4·09–17·99%)	6·72% (3·50–11·25%)	4·69% (2·21–8·31%)	6·06% (3·35–9·39%)	11·15% (6·92–16·73%)
West Bank and Gaza	PSE	EMR	LMIC	17·55% (11·02–25·37%)	3·84% (1·21–8·56%)	2·72% (0·99–5·47%)	1·79% (0·64–3·61%)	2·65% (1·18–4·95%)	5·71% (3·01–9·59%)
Russian Federation	RUS	EUR	UMIC	21·05% (9·71–35·06%)	5·72% (1·03–16·36%)	3·98% (0·79–10·15%)	2·72% (0·53–7·53%)	3·72% (0·98–8·88%)	7·44% (2·61–15·60%)
Rwanda	RWA	AFR	LIC	34·00% (23·66–45·19%)	13·27% (5·49–24·48%)	9·38% (4·22–16·80%)	6·70% (2·87–12·18%)	8·22% (4·20–14·13%)	14·31% (8·09–22·56%)
Sudan	SDN	EMR	LIC	38·28% (30·12–46·80%)	16·47% (8·04–27·47%)	11·67% (6·53–18·14%)	8·47% (4·54–13·54%)	10·09% (6·33–14·61%)	17·00% (11·11–24·11%)
Senegal	SEN	AFR	LMIC	24·95% (17·69–32·98%)	7·33% (2·97–14·40%)	5·15% (2·34–9·01%)	3·52% (1·55–6·18%)	4·74% (2·61–7·82%)	9·14% (5·43–14·08%)
Solomon Islands	SLB	WPR	LMIC	23·59% (15·44–32·83%)	6·65% (2·31–13·90%)	4·69% (1·89–8·82%)	3·19% (1·19–6·16%)	4·34% (2·07–7·92%)	8·49% (4·58–13·93%)
Sierra Leone	SLE	AFR	LIC	51·55% (37·28–66·71%)	29·45% (12·92–50·91%)	21·58% (10·16–37·32%)	16·68% (7·12–30·52%)	18·10% (8·91–30·20%)	27·47% (15·28–43·13%)
El Salvador	SLV	AMR	LMIC	32·79% (20·80–45·05%)	12·43% (4·44–24·26%)	8·81% (3·58–17·03%)	6·28% (2·33–12·50%)	7·77% (3·30–14·29%)	13·62% (6·89–22·63%)
Somalia	SOM	EMR	LIC	58·83% (47·99–68·99%)	37·63% (22·24–56·59%)	28·00% (16·48–40·20%)	22·19% (12·58–33·46%)	23·30% (15·07–32·95%)	33·99% (22·87–46·44%)
Serbia	SRB	EUR	UMIC	42·04% (19·80–65·92%)	20·85% (4·15–48·40%)	15·17% (3·07–35·84%)	11·46% (2·09–29·92%)	12·96% (2·99–30·09%)	20·50% (6·42–42·03%)
South Sudan	SSD	AFR	LIC	36·66% (28·66–45·58%)	15·17% (7·34–26·03%)	10·73% (5·72–17·02%)	7·74% (3·98–12·66%)	9·33% (5·64–13·86%)	15·93% (10·27–23·03%)
São Tomé and Principe	STP	AFR	LMIC	44·30% (31·84–57·69%)	21·95% (9·53–38·29%)	15·78% (7·52–26·62%)	11·82% (5·53–21·18%)	13·45% (6·75–22·71%)	21·46% (12·23–33·30%)
Suriname	SUR	AMR	UMIC	49·76% (37·00–62·87%)	27·42% (13·01–45·86%)	19·93% (9·94–32·95%)	15·28% (7·16–26·85%)	16·78% (9·01–26·61%)	25·83% (15·22–39·03%)
Swaziland	SWZ	AFR	LMIC	34·75% (25·86–44·90%)	13·77% (6·08–25·20%)	9·71% (4·83–15·89%)	6·98% (3·22–12·44%)	8·51% (4·70–13·46%)	14·74% (8·94–22·38%)
Syrian Arab Republic	SYR	EMR	LIC	27·95% (18·80–38·71%)	9·24% (3·27–18·29%)	6·47% (2·67–12·59%)	4·50% (1·64–8·87%)	5·81% (2·90–9·99%)	10·80% (5·85–17·69%)
Chad	TCD	AFR	LIC	43·93% (28·71–59·82%)	21·91% (7·95–42·53%)	15·72% (6·11–29·23%)	11·82% (4·27–23·23%)	13·34% (5·91–23·78%)	21·35% (10·59–35·72%)
Togo	TGO	AFR	LIC	30·83% (21·02–41·73%)	11·05% (4·32–21·01%)	7·78% (3·47–14·12%)	5·48% (2·23–10·47%)	6·90% (3·50–11·84%)	12·41% (6·91–19·84%)

Country	Code	Region	Income level	Poorest	Poorer	Middle	Richer	Richest	Overall
Thailand	THA	SEAR	UMIC	13·94% (8·56–20·88%)	2·54% (0·66–6·35%)	1·79% (0·61–3·79%)	1·16% (0·35–2·58%)	1·82% (0·77–3·42%)	4·25% (2·19–7·40%)
Tajikistan	TJK	EUR	LMIC	55·03% (43·12–66·21%)	33·14% (17·88–51·92%)	24·39% (13·54–37·29%)	19·07% (9·99–30·40%)	20·37% (12·42–30·27%)	30·40% (19·39–43·22%)
Turkmenistan	TKM	EUR	UMIC	24·39% (14·00–36·29%)	7·18% (1·89–16·45%)	5·09% (1·61–10·76%)	3·49% (0·99–8·00%)	4·68% (1·65–9·56%)	8·96% (4·03–16·21%)
Timor-Leste	TLS	SEAR	LMIC	34·46% (20·42–51·03%)	13·88% (3·87–29·57%)	9·88% (3·08–21·22%)	7·13% (2·10–16·43%)	8·64% (3·12–17·40%)	14·80% (6·52–27·13%)
Tonga	TON	WPR	UMIC	18·43% (11·81–25·98%)	4·19% (1·36–9·21%)	2·96% (1·15–5·75%)	1·96% (0·70–3·89%)	2·87% (1·32–5·22%)	6·08% (3·27–10·01%)
Tunisia	TUN	EMR	LMIC	31·76% (20·42–44·32%)	11·70% (4·14–22·90%)	8·28% (3·36–15·89%)	5·87% (2·19–11·51%)	7·34% (3·21–13·20%)	12·99% (6·66–21·57%)
Turkey	TUR	EUR	UMIC	16·37% (11·13–22·67%)	3·34% (1·08–7·23%)	2·36% (1·00–4·27%)	1·54% (0·60–2·93%)	2·35% (1·22–3·90%)	5·19% (3·01–8·20%)
Tuvalu	TUV	WPR	UMIC	28·41% (14·84–46·08%)	9·84% (2·09–25·31%)	6·98% (1·71–16·90%)	4·92% (1·09–12·65%)	6·24% (1·80–13·69%)	11·28% (4·31–22·93%)
Tanzania, United Republic of	TZA	AFR	LMIC	27·07% (19·83–34·90%)	8·54% (3·73–16·43%)	6·00% (2·95–10·08%)	4·14% (1·92–7·18%)	5·45% (3·09–8·55%)	10·24% (6·30–15·43%)
Uganda	UGA	AFR	LIC	28·53% (21·34–36·49%)	9·47% (3·94–17·96%)	6·63% (3·50–11·18%)	4·61% (2·17–7·95%)	5·96% (3·67–8·95%)	11·04% (6·92–16·51%)
Ukraine	UKR	EUR	LMIC	40·84% (28·92–53·16%)	18·88% (7·96–34·81%)	13·44% (6·13–23·61%)	9·95% (4·01–18·05%)	11·53% (5·93–19·42%)	18·93% (10·59–29·81%)
Uzbekistan	UZB	EUR	LMIC	43·94% (30·31–56·70%)	21·73% (8·99–39·71%)	15·56% (6·80–26·66%)	11·67% (4·80–20·66%)	13·24% (6·61–22·00%)	21·23% (11·50–33·15%)
St· Vincent and the Grenadines	VCT	AMR	UMIC	13·54% (8·43–19·81%)	2·36% (0·72–5·46%)	1·69% (0·58–3·38%)	1·08% (0·37–2·21%)	1·74% (0·77–3·22%)	4·08% (2·17–6·82%)
Venezuela	VEN	AMR	UMIC	15·00% (9·62–21·27%)	2·83% (0·95–6·42%)	2·02% (0·80–3·98%)	1·31% (0·52–2·65%)	2·05% (0·95–3·65%)	4·64% (2·57–7·59%)
Vietnam	VNM	WPR	LMIC	32·90% (19·94–46·85%)	12·72% (4·04–27·30%)	8·94% (2·97–18·11%)	6·43% (2·04–13·40%)	7·86% (3·17–15·23%)	13·77% (6·43–24·18%)
Vanuatu	VUT	WPR	LMIC	16·91% (10·90–24·07%)	3·56% (1·14–7·94%)	2·52% (0·98–4·84%)	1·65% (0·60–3·20%)	2·48% (1·17–4·44%)	5·42% (2·96–8·90%)
Samoa	WSM	WPR	LMIC	19·92% (12·25–29·18%)	4·88% (1·50–10·86%)	3·44% (1·22–7·08%)	2·30% (0·78–4·76%)	3·28% (1·37–6·20%)	6·76% (3·42–11·62%)
Kosovo	XKX	EUR	UMIC	30·51% (19·31–42·19%)	10·84% (3·73–21·54%)	7·68% (3·07–15·03%)	5·42% (1·97–10·91%)	6·84% (2·88–12·71%)	12·26% (6·19–20·48%)
Yemen	YEM	EMR	LIC	35·20% (26·67–44·34%)	14·07% (6·47–24·72%)	9·92% (5·29–16·29%)	7·11% (3·61–11·70%)	8·67% (5·22–13·17%)	14·99% (9·45–22·04%)

Country	Code	Region	Income level	Poorest	Poorer	Middle	Richer	Richest	Overall
South Africa	ZAF	AFR	UMIC	33·24% (19·42–49·55%)	13·01% (3·40–28·68%)	9·23% (2·84–20·43%)	6·65% (1·92–16·12%)	8·12% (2·80–16·35%)	14·05% (6·08–26·23%)
Zambia	ZMB	AFR	LIC	31·95% (22·61–42·58%)	11·84% (4·73–22·97%)	8·29% (3·68–14·59%)	5·90% (2·47–10·96%)	7·34% (3·96–12·16%)	13·06% (7·49–20·65%)
Zimbabwe	ZWE	AFR	LMIC	43·29% (30·02–56·88%)	21·08% (8·57–37·95%)	15·15% (6·66–27·22%)	11·30% (4·77–21·01%)	12·93% (5·98–22·78%)	20·75% (11·20–33·17%)

* All countries include 135 low- and middle-income countries analyzed. Values in parentheses represent equal-tailed 95% credible intervals.

Figure S14. Comparison of predicted costs and World Health Organization Tuberculosis Patient Cost Survey (TB-PCS) data for direct medical costs (DM), direct non-medical costs (NM), and indirect costs (IND), stratified by rifampicin-sensitive (RS) and rifampicin-resistant (RR) TB.

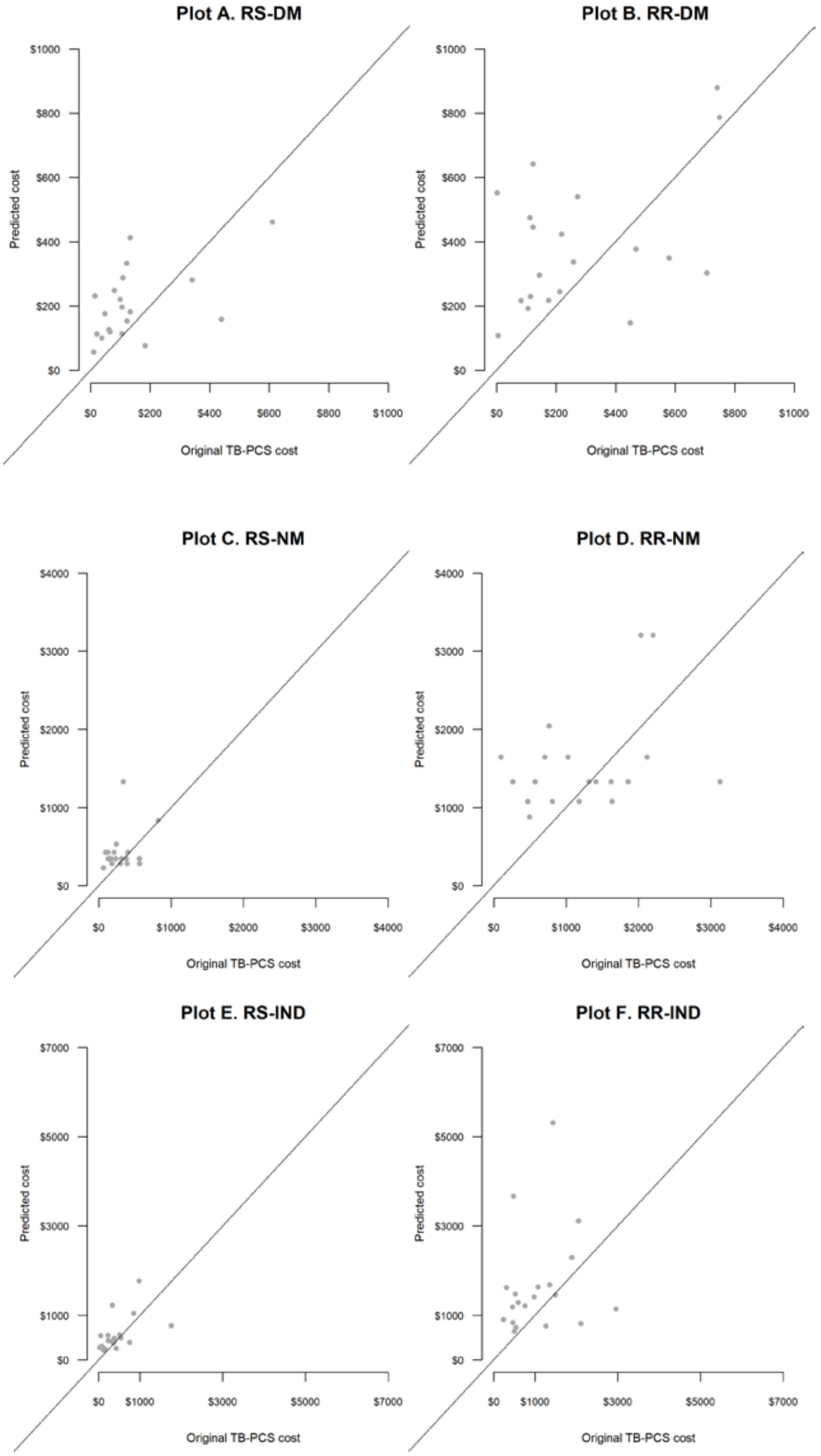

Figure S15. Comparison of residuals vs. model fit for direct medical costs (DM), direct non-medical costs (NM), and indirect costs (IND).

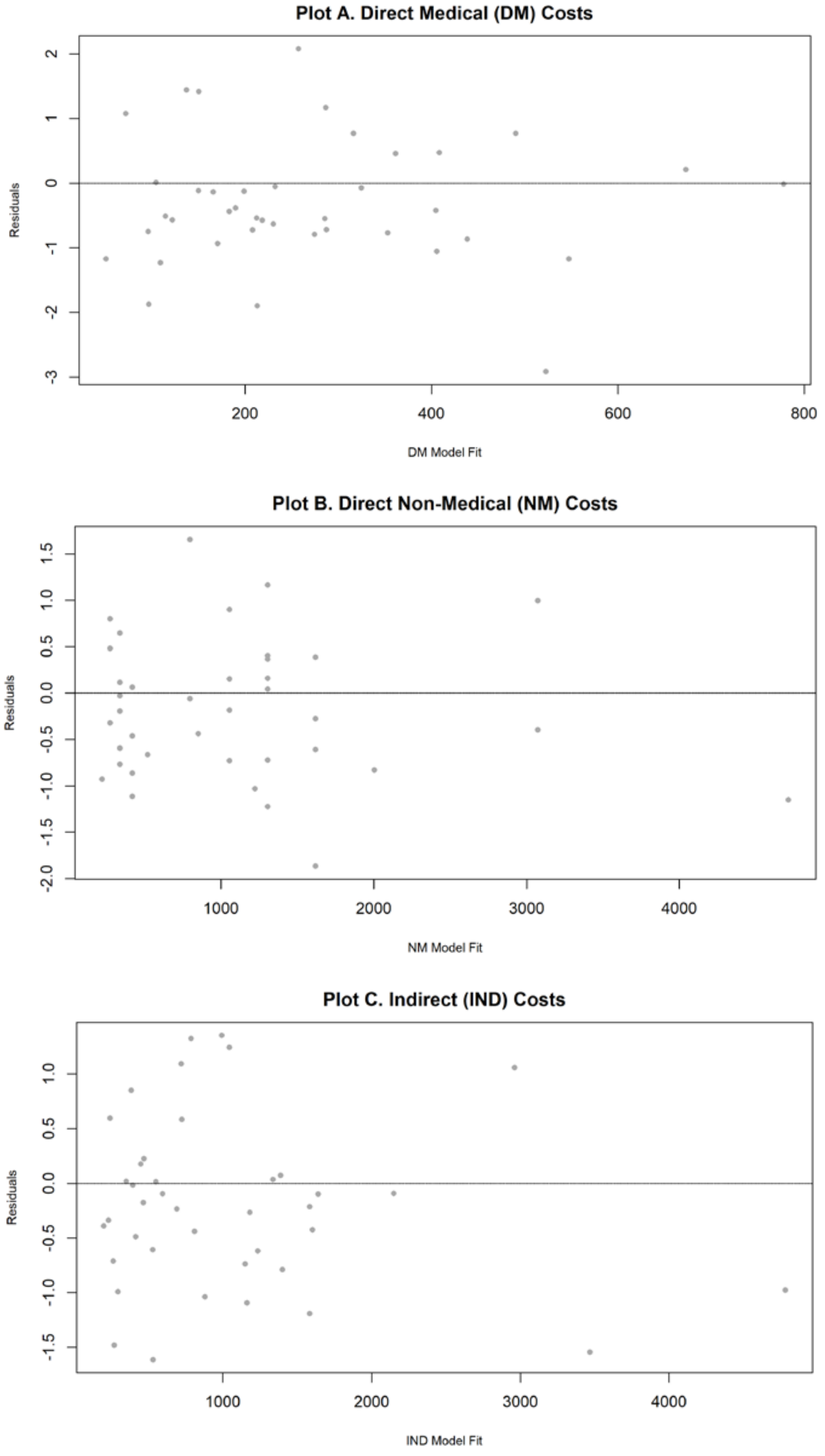

Table S16. Comparison of World Health Organization Tuberculosis Patient Cost Survey (TB-PCS) data to predicted patient costs by country and drug resistance category (RR = rifampicin-resistant; RS = rifampicin-sensitive) in 2021 USD.

		Direct medical costs			
		RS		RR	
Country	Code	TB-PCS	Prediction	TB-PCS	Prediction
Congo, Democratic Republic of the	COD	65·47	119·09	115·36	228·88
Kenya	KEN	56·30	175·90	300·58	337·73
Mali	MLI	474·44	157·73	761·92	302·55
Thailand	THA	105·28	113·33	174·01	217·80
Tanzania, United Republic of	TZA	40·68	99·84	113·94	192·09
Uganda	UGA	23·19	112·84	91·06	216·95
		**Direct non-medical costs**			
		**RS**		**RR**	
**Country**	**Code**	**TB-PCS**	**Prediction**	**TB-PCS**	**Prediction**
Congo, Democratic Republic of the	COD	174·40	345·66	574·82	1328·11
Kenya	KEN	152·96	427·87	821·64	1644·11
Mali	MLI	606·67	345·66	1521·48	1328·11
Thailand	THA	379·09	345·66	1839·26	1328·11
Tanzania, United Republic of	TZA	140·60	345·66	282·45	1328·11
Uganda	UGA	175·13	345·66	3462·23	1328·11
		**Indirect costs**			

Country	Code	RS		RR	
		TB-PCS	Prediction	TB-PCS	Prediction
Congo, Democratic Republic of the	COD	161·72	241·68	540·13	723·33
Kenya	KEN	59·10	539·03	359·43	1616·10
Mali	MLI	38·35	278·82	502·68	834·71
Thailand	THA	355·91	381·01	2927·22	1138·24
Tanzania, United Republic of	TZA	89·80	301·67	250·89	903·23
Uganda	UGA	117·41	271·75	2334·26	813·40

Table S17. Comparison of World Health Organization Tuberculosis Patient Cost Survey (TB-PCS) data to predicted proportions of households experiencing catastrophic costs by country and income quintile.

Country	Code	Income measure	Quintile	TB-PCS catastrophic cost proportion	Predicted catastrophic cost proportion
Congo, Democratic Republic of the	COD	expenditure	Poorest	76·82% (68·13–84·49%)	80·90% (75·87–86·2%)
			Poorer	65·27% (55·42–74·5%)	69·77% (58·91–79·34%)
			Middle	61·09% (50·98–70·75%)	61·75% (50·41–73·67%)
			Richer	44·35% (34·92–53·98%)	57·27% (44·87–69·79%)
			Richest	34·31% (24·30–45·09%)	51·13% (40·83–61·95%)
			Overall	56·49% (50·12–62·75%)	64·16% (54·18–74·19%)
Kenya	KEN	expenditure	Poorest	56·41% (48·75–63·92%)	84·45% (81·04–87·09%)
			Poorer	24·86% (16·94–33·74%)	75·23% (70·54–79·94%)
			Middle	18·97% (12·45–26·49%)	67·46% (60·78–73·79%)
			Richer	13·22% (8·10–19·37%)	62·06% (53·19–70·17%)
			Richest	10·10% (5·28–16·24%)	54·64% (47·11–62·47%)
			Overall	26·51% (20·75–32·27%)	68·77% (62·53–74·69%)
Mali	MLI	income	Poorest	84·21% (76·15–90·87%)	79·37% (74·50–84·24%)
			Poorer	66·67% (53·94–78·27%)	65·45% (56·37–73·83%)
			Middle	43·09% (29·75–56·96%)	56·00% (46·48–66·61%)
			Richer	32·76% (17·33–50·40%)	49·72% (39·04–60·25%)
			Richest	15·56% (7·46–25·93%)	45·27% (36·20–54·88%)
			Overall	49·45% (40·68–58·23%)	59·16% (50·52–67·96%)
Thailand	THA	expenditure	Poorest	67·99% (58·38–76·89%)	60·70% (54·11–66·87%)
			Poorer	36·38% (29·97–43·04%)	37·17% (27·39–49·21%)
			Middle	20·88% (15·65–26·65%)	28·20% (18·78–39·10%)

Country	Code	Income measure	Quintile	TB-PCS catastrophic cost proportion	Predicted catastrophic cost proportion
			Richer	14·07% (9·70–19·10%)	22·67% (13·84–33·51%)
			Richest	10·00% (6·46–14·21%)	23·81% (15·85–33·66%)
			Overall	29·89% (25·57–34·39%)	34·51% (25·99–44·47%)
Tanzania, United Republic of	TZA	expenditure	Poorest	87·12% (79·58–93·13%)	81·2% (77·81–84·61%)
			Poorer	62·08% (53·47–70·32%)	69·3% (62·64–75·54%)
			Middle	38·58% (28·61–49·05%)	60·9% (52·71–69·02%)
			Richer	25·18% (17·54–33·68%)	54·98% (45·61–64·56%)
			Richest	10·95% (5·90–17·32%)	48·97% (40·17–57·79%)
			Overall	44·88% (36·83–53·07%)	63·07% (55·79–70·30%)
Uganda	UGA	income	Poorest	85·63% (76·85–92·60%)	79·91% (75·72–84·12%)
			Poorer	65·68% (55·99–74·76%)	67·08% (58·60–74·83%)
			Middle	54·18% (43·97–64·22%)	58·35% (49·08–68·13%)
			Richer	39·38% (28·26–51·08%)	52·62% (42·33–63·32%)
			Richest	26·14% (20·66–32·02%)	47·32% (38·44–56·76%)
			Overall	53·12% (42·75–63·36%)	61·06% (52·83–69·43%)
